# Assessment of bacterial and structural dynamics in aerobic granular biofilms

**DOI:** 10.3389/fmicb.2013.00175

**Published:** 2013-07-10

**Authors:** David G. Weissbrodt, Thomas R. Neu, Ute Kuhlicke, Yoan Rappaz, Christof Holliger

**Affiliations:** ^1^Laboratory for Environmental Biotechnology, School for Architecture, Civil and Environmental Engineering, Ecole Polytechnique Fédérale de LausanneLausanne, Switzerland; ^2^Institute of Environmental Engineering, ETH ZurichZurich, Switzerland; ^3^Department of Process EngineeringEawag, Duebendorf, Switzerland; ^4^Microbiology of Interfaces, Department of River Ecology, Helmholtz Centre for Environmental Research - UFZMagdeburg, Germany

**Keywords:** biological wastewater treatment, aerobic granular sludge, granular biofilm formation and structure, T-RFLP, pyrosequencing, CLSM, FISH, FLBA

## Abstract

Aerobic granular sludge (AGS) is based on self-granulated flocs forming mobile biofilms with a gel-like consistence. Bacterial and structural dynamics from flocs to granules were followed in anaerobic-aerobic sequencing batch reactors (SBR) fed with synthetic wastewater, namely a bubble column (BC-SBR) operated under wash-out conditions for fast granulation, and two stirred-tank enrichments of *Accumulibacter* (PAO-SBR) and *Competibacter* (GAO-SBR) operated at steady-state. In the BC-SBR, granules formed within 2 weeks by swelling of *Zoogloea* colonies around flocs, developing subsequently smooth zoogloeal biofilms. However, *Zoogloea* predominance (37–79%) led to deteriorated nutrient removal during the first months of reactor operation. Upon maturation, improved nitrification (80–100%), nitrogen removal (43–83%), and high but unstable dephosphatation (75–100%) were obtained. Proliferation of dense clusters of nitrifiers, *Accumulibacter*, and *Competibacter* from granule cores outwards resulted in heterogeneous bioaggregates, inside which only low abundance *Zoogloea* (<5%) were detected in biofilm interstices. The presence of different extracellular glycoconjugates detected by fluorescence lectin-binding analysis showed the complex nature of the intracellular matrix of these granules. In the PAO-SBR, granulation occurred within two months with abundant and active *Accumulibacter* populations (56 ± 10%) that were selected under full anaerobic uptake of volatile fatty acids and that aggregated as dense clusters within heterogeneous granules. Flocs self-granulated in the GAO-SBR after 480 days during a period of over-aeration caused by biofilm growth on the oxygen sensor. Granules were dominated by heterogeneous clusters of *Competibacter* (37 ± 11%). *Zoogloea* were never abundant in biomass of both PAO- and GAO-SBRs. This study showed that *Zoogloea, Accumulibacter*, and *Competibacter* affiliates can form granules, and that the granulation mechanisms rely on the dominant population involved.

## Introduction

Conventional activated sludge systems operated for biological nutrient removal (BNR) from wastewater require a high footprint for the integration of activated sludge tanks enabling microbial processes and of secondary clarifiers for the separation of biomass from the treated effluent. The aerobic granular sludge (AGS) process has recently deserved the attention of innovation analysts (Radauer et al., 2012). This technology has been developed for intensified BNR and secondary clarification in single sequencing batch reactors (SBR), and is related to definite savings in land area, construction, and operation costs according to reports on the performance of first full-scale plants (Giesen et al., 2012; Inocencio et al., 2013). AGS comprises suspended biofilm particles, called aerobic granules, formed by self-aggregation of microbial populations (Morgenroth et al., 1997). Although some full-scale plants are getting installed world-wide, the granulation mechanism at the microbial community level is not yet fully understood and improved knowledge of this phenomenon may enable further process optimization.

Nutrient removal deficiency and other process instabilities during granulation have been observed in several studies (Morgenroth et al., 1997; de Kreuk et al., 2005; Liu and Liu, 2006; Gonzalez-Gil and Holliger, 2011). Wash-out conditions that have been recommended for the selection of fast-settling biomass (Beun et al., 1999) have been shown to result in the deterioration of the settling properties and of the BNR performances caused by bacterial community imbalances with overgrowth of filamentous or zoogloeal populations, respectively (Weissbrodt et al., 2012a). Nevertheless, granule formation has been positively correlated with proliferation of *Zoogloea* spp. under wash-out conditions (Etterer, 2006; Adav et al., 2009; Ebrahimi et al., 2010; Weissbrodt et al., 2012a). Although *Zoogloea* can produce cohesive extracellular polymeric substances (EPS) (Seviour et al., 2012), it remains unclear whether these organisms are required to initiate granulation. Shifts in predominant populations in AGS systems have further been related to specific operation parameters. For instance, nutrient composition, temperature, carbon source, and selective excess sludge removal can impact the competition of polyphosphate-(PAO) and glycogen-accumulating organisms (GAO) related to “*Candidati* Accumulibacter and Competibacter phosphates” (hereafter referred to as *Accumulibacter* and *Competibacter*), respectively (de Kreuk and van Loosdrecht, 2004; Ebrahimi et al., 2010; Gonzalez-Gil and Holliger, 2011; Winkler et al., 2011; Bassin et al., 2012). Since some clades have been reported to denitrify, PAO and GAO can in addition be impacted by the type of terminal electron acceptor present in the medium (Kuba et al., 1997; Oehmen et al., 2010).

Granule structures comprise stratification of microbial niches oriented along radial substrate and microhabitat gradients (de Kreuk et al., 2005). Whereas bacterial community dynamics at reactor scale (Liu et al., 1998) can be assessed by terminal-restriction fragment length polymorphism (T-RFLP) analysis, biofilm architecture and microbial arrangements can be examined by confocal laser scanning microscopy (CLSM) combined with fluorescence *in situ* hybridization (FISH) (Wagner et al., 1994; Wuertz et al., 2004; Nielsen et al., 2009; Neu et al., 2010). Localization of bacterial populations along dissolved oxygen (DO) gradients in granules has been investigated with the latter methods (Tsuneda et al., 2003; Kishida et al., 2006; Lemaire et al., 2008a; Yilmaz et al., 2008; Gao et al., 2010; Filali et al., 2012).

In a lab-scale SBR operated for simultaneous nitrification, denitrification and phosphorus removal, Lemaire et al. (2008b) have for instance found that *Accumulibacter* and *Competibacter* have been predominant in the underlying bacterial community. According to FISH and oxygen microsensor measurements, *Accumulibacter* have dominated the oxygenated 200-μm outer layer of granules, while *Competibacter* have been more abundant in the anoxic granule core. However, among all studies having investigated microbial stratification within granules, it has been found that ammonium-oxidizing organisms (AOO) have formed clusters near the surface, but no consensus has been found on the position of PAO and GAO within the granules and their involvement in denitrification.

Since granules are biofilms, they are likely to exhibit complex spatial architecture depending on specific process conditions (Lawrence et al., 1996; Okabe et al., 1997; Alpkvist and Klapper, 2007). Granular biofilms have been composed of specific matrices of exopolymeric substances such as “granulan” (Seviour et al., 2011) or alginate (Lin et al., 2010). The combination of fluorescence lectin-binding analysis (FLBA) and CLSM (Neu and Lawrence, 1999; Staudt et al., 2003) could be relevant for mapping glycoconjugates in the granular biofilm matrix. Such analysis has previously been conducted for the staining of global exopolysaccharide matrices in different types of granules (Tay et al., 2003; McSwain et al., 2005; Adav et al., 2008). Since lectins display specific binding properties, a screening of different lectins can provide additional information on the type of polysaccharide residues present in granular biofilms.

The present research was conducted to elucidate the dynamics of the bacterial communities and of the structures of bioaggregates during transitions from activated sludge flocs to early-stage nuclei and to mature granular biofilms. Granulation was studied in one bubble-column (BC-SBR) and two stirred-tank (PAO-SBR, GAO-SBR) anaerobic-aerobic SBRs operated under conditions selecting for fast AGS cultivation and for enrichments of *Accumulibacter* and *Competibacter* in activated sludge, respectively. T-RFLP, pyrosequencing, CLSM, FLBA, and FISH methods were combined to investigate the mechanisms of bacterial selection, granule formation, and biofilm maturation in relation with the evolution of process variables.

## Materials and methods

### Bubble-column SBR operation under wash-out dynamics

The BC-SBR was operated at 23 ± 2°C under wash-out conditions as reported in Weissbrodt et al. (2012a). This 2.5-L single-wall PVC reactor (height-to-diameter H/D ratio of 28) was inoculated with 3 g_VSS_ L^−1^ of activated sludge from a BNR wastewater treatment plant (WWTP Thunersee, Switzerland). The fixed 3-h SBR cycles comprised anaerobic feeding (60 min), aeration (110 min), settling (stepwise decrease from 15 to 3 min), and withdrawal (remaining cycle time; volume exchange ratio of 50%). Wash-out was generated by short settling and hydraulic retention times (HRT, 6 h). The sludge retention time (SRT) was not controlled. The synthetic wastewater composition comprised 4.8 _gP-PO4_ and 12.5 _gN-NH4_ per 100 g_CODs_ of acetate (Supplementary material 1). The system was fed over 220 days with a constant volumetric organic loading rate (OLR, 250 mg_CODs_ cycle^−1^ L^−1^_R_). The biomass specific OLR was initially 50 mg_CODs_ cycle^−1^ g^−1^_CODx_ and depended on the biomass amount remaining in the reactor. Aeration phases were run with up-flow superficial gas velocities of 0.025 m s^−1^, free DO evolution up to saturation, and pH 7.0 ± 0.2. Temperature, DO, pH, and electrical conductivity were recorded on-line.

### Stirred-tank PAO-SBR and GAO-SBR operation under steady-state

The PAO- and GAO-SBRs were run to cultivate activated sludge enrichments over 1–2 years. The 2.5-L double-wall glass reactors (Applikon Biotechnology, The Netherlands, H/D = 1.3) were inoculated with 3 g_VSS_ L^−1^ of BNR activated sludge, and operated according to Lopez-Vazquez et al. (2009b). SBR cycles comprised N_2_-flush (7 min), pulse feeding (7.3 min), N_2_-flush (5 min), anaerobic, aerobic and settling phases (for timing see below), and withdrawal (5 min; 50%). During the anaerobic and aerobic (3.5 ± 0.5 mg_O2_ L^−1^) phases, the reactor content was stirred at 300 rpm. Nitrification was inhibited in both reactors by addition of allyl-N-thiourea (Supplementary material 1). The SRTs were controlled by purge of excess sludge after aeration.

The PAO-SBR was operated at 17°C and pH 7.0–8.0, with 12-h HRT and 8-days SRT at steady-state, and with propionate, as well as with 9 g_CODs_ g^−1^_P−PO4_ in the influent wastewater following Schuler and Jenkins (2003). Enhanced anaerobic propionate uptake and orthophosphate-cycling activities were ensured by stepwise adaptation of the volumetric OLR from 15 to 200 mg_CODs_ cycle^−1^ L^−1^_R_ in 12 days, and by proper control of the anaerobic and aerobic contact times (3–5 h) based on on-line conductivity profiles (Maurer and Gujer, 1995; Aguado et al., 2006). Since fast-settling biomass formed after 30 days, the settling time was decreased from 60 to 10 min to save cycle time, and to prevent prolonged endogenous respiration.

The GAO-SBR was operated at 30°C and pH 6.5 ± 0.2, with 12-h HRT and longer 16-days SRT at steady-state (Lopez-Vazquez et al., 2009a), acetate, and 200 g_COD_ g^−1^_P−PO4_. The volumetric OLR, anaerobic phase length, and settling time were fixed at 200 mg_CODs_ cycle^−1^ L^−1^_R_, 3 h, and 60 min since start-up, respectively.

Schemes of experimental set-ups are available in Supplementary material 2. Each bubble-column and stirred-tank SBR experiment was operated on the long run. The definition of the operation conditions of the PAO- and GAO-SBRs nevertheless resulted from step-wise optimization based on multiple reactors experiments (Weissbrodt, 2012).

### Analyses of soluble compounds and biomass

Concentrations of volatile fatty acids (VFA) and inorganic ions were measured by high performance liquid chromatography and ion chromatography, respectively. Sludge compositions were characterized as fractions of total (TSS), volatile (VSS), and inorganic suspended solids (ISS). Details on analytical methods are available in open access elsewhere (Weissbrodt et al., 2012a).

### Molecular analyses of bacterial community compositions

Bacterial community dynamics were investigated by T-RFLP analysis (Weissbrodt et al., 2012a). Biomass samples were homogenized by grinding, aliquoted in 1.5-mL Eppendorf tubes, and stored at −20°C. Eubacterial 16S rRNA gene pools were targeted and amplified by PCR with the labeled 8f (FAM-5′-AGAGTTTGATCMTGGCTCAG-3′) and unlabeled 518r (5′-ATTACCGCGGCTGCTGG-3′) primers. Amplicons were digested with *Hae*III. T-RFLP profiles were generated with predominant operational taxonomic units (OTU) >2%. Richness and Shannon's H' diversity indices were computed in R in order to assess the impact of operation conditions on the overall bacterial community structures (R Development Core Team, 2008). Three biomass samples were analyzed in triplicates, leading to a relative standard deviation of 6% on the T-RFLP method.

OTUs were affiliated to phylotypes with the pyrosequencing-based PyroTRF-ID bioinformatics methodology (Weissbrodt et al., 2012b) applied to biomass grab samples collected on days 2 and 59 (BC-SBR), 109 (PAO-SBR), and 398 (GAO-SBR). The DNA extracts of these samples were sent to Research and Testing Laboratory LLC (Lubbock, TX, USA) for pyrosequencing analysis according to the protocol developed by the company (Sun et al., 2011). The pyrosequencing datasets underwent denoising, mapping, and *in silico* restriction in the PyroTRF-ID pipeline. Bacterial affiliations of OTUs were obtained by cross-correlating digital and experimental T-RFLP profiles. Greengenes (DeSantis et al., 2006) was used as mapping database in the PyroTRF-ID pipeline. The pyrosequencing dataset of a mature AGS sample (BC-II) originating from a precedent reactor operated under similar conditions was used to affiliate OTUs at later stage in the BC-SBR. Technical and biological replicates previously measured in Weissbrodt et al. (2012b) demonstrated that the pyrosequencing-based PyroTRF-ID method is reliable.

In order to obtain estimates of the evolution of the mass of target OTUs, biomass equivalents were calculated by multiplying the relative abundances of these OTUs with the VSS concentration, according to Weissbrodt et al. (2012a). It should nevertheless be pointed out that such calculations are only very rough estimations since the number of 16S rRNA copies per cell can range from 1 to 15 depending on the genome and that cell biomass vary between bacterial species (Kembel et al., 2012). However, this simplified mass-based approach can provide from an engineering point of view a deeper insight in the dynamics of target populations by complementing relative abundance data. This is particularly meaningful under the high biomass dynamics generated by operation under wash-out conditions.

### Confocal laser scanning microscopy analyses of flocs and granules

Structural transitions from flocs to granules were examined with CLSM. Collected biomass samples were washed twice in phosphate buffer saline (PBS) pH 7.4, and stored at 0–5°C in paraformaldehyde 4% (m/v in PBS). Fluorescent dyes were screened for mapping bioaggregates (Supplementary material 3). Rhodamine 6G was optimal for time series. Glycoconjugates were detected in selected AGS samples of the BC-SBR by FLBA, according to Staudt et al. (2003) and Zippel and Neu (2011). Spatial bacterial dynamics were followed by FISH-CLSM using rRNA oligonucleotide probes selected from probeBase (Loy et al., 2003) and targeting *Zoogloea*, *Accumulibacter* and *Competibacter* (BC-SBR), *Accumulibacter* and *Zoogloea* (PAO-SBR), as well as *Competibacter* (GAO-SBR) (Supplementary material 4). Samples were hybridized according to Nielsen et al. (2009). Granules smaller than 2 mm were cross-sectioned with a scalpel at ambient temperature in 0.5–1.0 mm deep CoverWell chambers mounted on microscopy slides (Life Technologies, Switzerland). Bigger granules were cryosectioned (80 μm) in a cryotome CM3050S (Leica, Germany) after freezing at −26°C in Tissue-Tek OCT compound (Sakura, The Netherlands).

The CLSM used was a TCS SP5X (Leica, Germany) equipped with upright microscope, an acusto optical beam splitter, and a supercontinuum light source. The system was controlled by the LAS AF software version 2.6.1. Samples were examined by the objective lenses 10 × 0.3 NA, 20 × 0.5 NA (overview), and 63 × 1.2 NA (high resolution). Excitation and emission wavelengths of fluorochromes are given in Supplementary materials 3 and 4. Each CLSM analysis was performed in at least two biological replicates. The CLSM reflection signal was collected as structural reference, and in order to distinguish voids from unstained regions. Multi-channel datasets were recorded in sequential mode to avoid cross-talk (Zippel and Neu, 2011). Images were collected by optical sample sectioning over thicknesses and stepsizes of 5–215 μm and 1–8 μm, respectively. Digital Leica Image Files (.lif) were processed in the Imaris software. Digital images were mainly represented as maximum intensity projections (MIP) with the Easy 3-D mode. Specific biofilm structures were examined in three dimensions either with the XYZ projection mode or the volume mode. The color intensity levels of the tagged image files generated in Imaris were slightly adjusted in the Photoshop software for improved resolution and contrast in the digital image datasets.

## Results

### Process and bacterial dynamics in the bubble-column SBR

**Figure 1:**
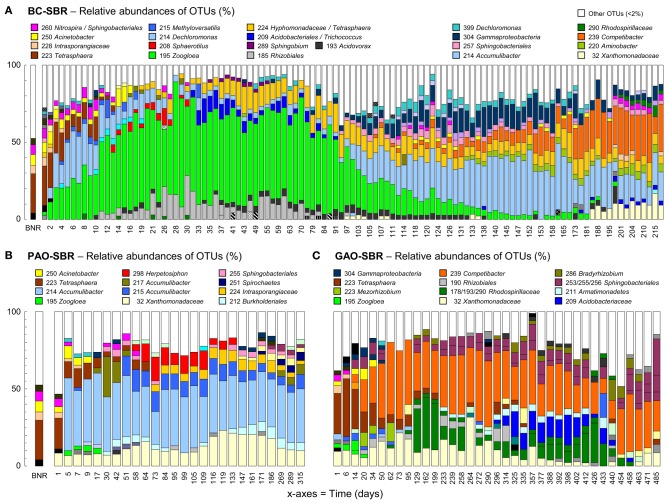
**Bacterial community dynamics in the BC-SBR (A), PAO-SBR (B), and GAO-SBR (C)**. Phylotypes were identified with the PyroTRF-ID methodology (Weissbrodt et al., 2012b) and were compiled for each reactor in Supplementary material 5. In the BC-SBR, *Dechloromonas* was mainly contributing to OTU-214 during the first 60 days, whereas *Accumulibacter* was mainly contributing to this OTU in the mature granular sludge.

**Figure 2:**
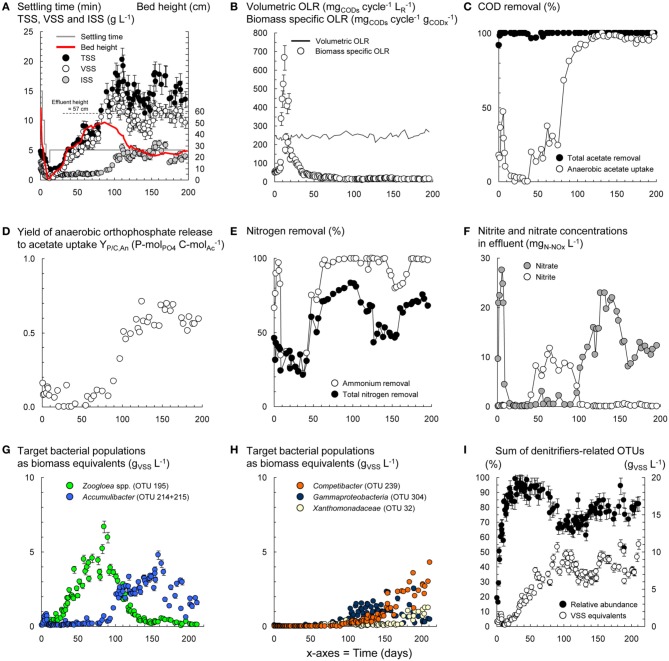
**Dynamics in process variables during the operation of the BC-SBR under wash-out conditions**. Evolution of the concentration and composition of biomass in terms of total (TSS), volatile (VSS), and inorganic suspended solids (ISS) during granule formation and maturation **(A)**. Variations of the biomass specific organic loading rate (OLR) during operation under wash-out conditions with constant volumetric OLR **(B)**. Dynamics in activities of COD removal **(C)**, orthophosphate-cycling **(D)**, nitrification and nitrogen removal **(E,F)**. Shifts in predominant bacterial populations during granule formation and maturation **(G,H)**. Rough estimation of the evolution of the sum of OTUs possibly affiliating with denitrifiers, according to the physiological properties reported in literature for the underlying phylotypes detected with PyroTRF-ID **(I)**.

The formation and maturation of granules in the BC-SBR operated under wash-out conditions were linked to dynamics in bacterial community compositions (Figure 1A and Supplementary material 5) and in process variables (Figure 2) over 220 days. The start-up period over the first 60 days was previously described in details in Weissbrodt et al. (2012a).

Wash-out conditions mainly impacted on the process before important accumulation of granular biomass. Under low residual biomass concentration, the operation with constant volumetric OLR and fixed anaerobic feeding phase length resulted in transiently high biomass specific OLR (Figures 2A–C). Leakage of VFA into aeration resulted in hampered BNR activities (Figures 2D–F) and selection of *Zoogloea* (OTU-195, 37–79%, 6.7 g_VSS_ L^−1^) over *Accumulibacter* (OTU-214) and *Competibacter* (OTU-239) affiliates (Figures 2G,H). After day 90, the shift from *Zoogloea* to *Accumulibacter* (33 ± 5%, 3.2 ± 0.6 g_VSS_ L^−1^) correlated with full acetate uptake and high apparent yield of orthophosphate release to acetate uptake (Y_P/C,An_ = 0.60 ± 0.07 P-mol C-mol^−1^_Ac_) under anaerobic conditions. Phosphorus was fully removed (data not shown) leading to 32 ± 1% of ISS in AGS. After day 110, the bed dropped from 40–50 to <30 cm together with a decrease in biomass from 20 to <15 g_TSS_ L^−1^ and spontaneous stabilization of the SRT at 24 ± 5 days (data not shown). Proliferation of *Competibacter* (20–30%, 3.6 g_VSS_ L^−1^) over *Accumulibacter* (10–15%, 0.6–1.9 g_VSS_ L^−1^) after 150 days correlated with lower Y_P/C,An_ (0.50 P-mol C-mol^−1^_Ac_) and instable dephosphatation (82 ± 25%).

Nitrification and nitrogen removal were inactive during wash-out, but recovered up to 90% and 70%, respectively, together with accumulation of AGS and lowering of the biomass specific OLR. Nitrite and then nitrate accumulated between days 50 and 150 (Figure 2E). Efficient nitrogen removal between days 50 and 100 (71–83%) and 150–200 (63–75%) correlated with high totals of denitrifiers-related OTUs (Figure 2I). Nitrifier-related OTUs were hardly detectable with T-RFLP. One OTU-260 affiliating with *Sphingobacteriales* and nitrite-oxidizing *Nitrospira* increased from 0.5 ± 0.4% (days 30–60) to 1.6 ± 0.7% (days 110–200). Further analysis of the pyrosequencing datasets in MG-RAST (Meyer et al., 2008) suggested shifts in low abundance ammonium- (AOO) and nitrite-oxidizing organisms (NOO) from flocculent sludge on day 2 (*Nitrosomonas* 0.12%, *Nitrosococcus* 0.24%, *Nitrosovibrio* 0.06%, *Nitrobacter* 0.06%, *Nitrospira* 1.02%) to early-stage AGS on day 59 (*Nitrosospira* 0.03%) and mature AGS in sample BC-II (*Nitrococcus* 6.2%, *Nitrosomonas* 0.1%, *Nitrospira* 0.2%).

### Process and bacterial dynamics in the stirred-tank PAO-SBR and GAO-SBR

**Figure 3:**
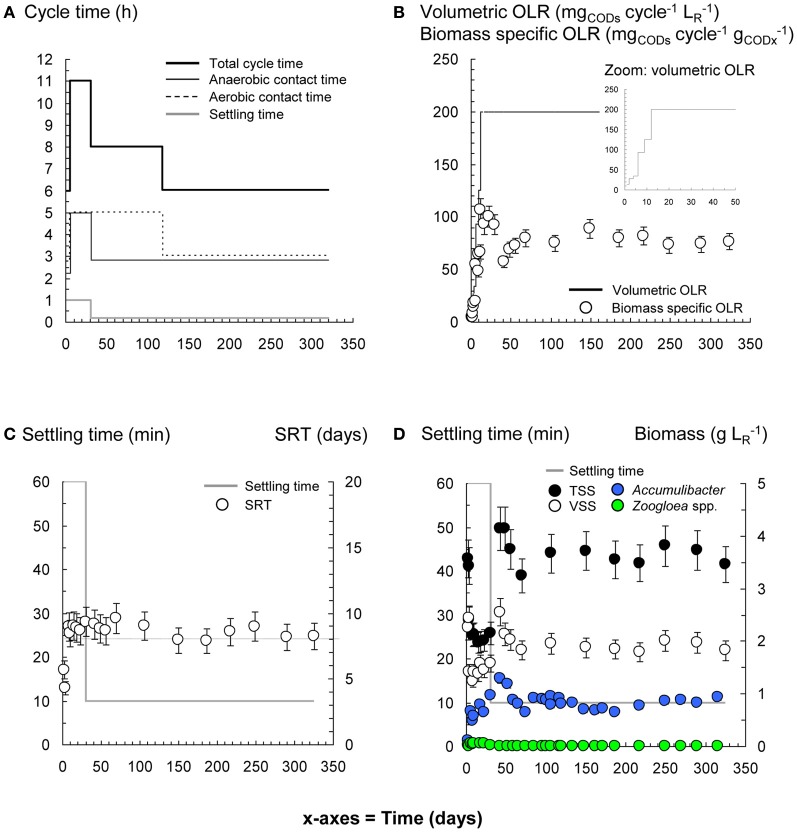
**Process and bacterial dynamics in the PAO-SBR**. The reactor was operated with proper control of anaerobic and aerobic contact times **(A)**, volumetric organic loading rate (OLR) **(B)**, and sludge retention time (SRT) **(C)** for rapid and preferential selection of *Accumulibacter* **(D)**.

The operation of the PAO-SBR under full control of the anaerobic contact time (5–3 h), volumetric OLR from 15 to 200 mg_CODs_ cycle^−1^ L^−1^_R_, SRT (8–10 days), and biomass concentration (>1.5 g_VSS_ L^−1^) resulted in the full uptake of propionate under anaerobic conditions, and in the rapid enrichment of *Accumulibacter* in the activated sludge (48% on day 5) (Figures 1B, 3). Enhanced orthophosphate-cycling activities were measured during the whole experimental period with Y_P/C,An_ ranging between 0.56 and 0.64 P-mol C-mol^−1^_Pr_. Steady-state was reached after 15 days, with gradual stabilization of the SRT from 5 to 8–10 days by purging excess sludge. Interestingly, fast-settling biomass nuclei (<500 μm) formed after 20 days. By decreasing the settling time from 60 to 10 min to save cycle time, nuclei evolved toward 1–2 mm large granules over the next 40 days. *Tetrasphaera* declined from 22% to 3% within 30 days, but remained between 2–10% in the system (Figure 1B). The enrichment displayed constant predominance of *Accumulibacter* (41 ± 6%), and significant level of *Xanthomonadaceae* (OTU-32, 7–20%) right from start-up. *Herpetosiphon* (OTU-298) amounted to 7–17% on days 60–120. *Zoogloea* was only detected up to day 20 with relative abundances below 4%.

In the GAO-SBR, biomass remained flocculent over more than 450 days. With constant volumetric OLR of 200 mg_CODs_ cycle^−1^ L^−1^_R_, full anaerobic acetate uptake was obtained from day 20 onward (data not shown). *Tetrasphaera* dominated over the first 2 weeks (31–47%), and remained at 5% abundance up to day 200 (Figure 1C). *Competibacter* prevailed in the enrichment (22–59%). Despite operation at steady-state, accompanying guilds displayed quite high dynamics. For instance abundances of *Alphaproteobacteria* related to *Rhodospirillaceae* (OTUs 178, 193, and 290, 2–40%), *Rhizobiales* (OTU-190, 2–12%), *Bradyrhizobium* (OTU-285, 2–8%), and *Sphingomonas* (OTU-287, 2–20%), as well as *Acidobacteriaceae* (OTU-209, 7–23%) and *Thiobacillus* relatives (OTU-216, 21% on day 433) fluctuated considerably during rector operation. After more than 450 days, fast-settling nuclei (<500 μm) were observed in the system, and evolved toward 1–2 mm granules from day 480 onward. Granulation correlated with transient over-aeration caused by biofilm growth on DO sensors. An increase in *Sphingobacteriales* relatives up to more than 40% (OTUs 253–256) was observed during granule formation and *Zoogloea*, *Accumulibacter*, and *Rhodocyclaceae* relatives were almost absent (<5%).

### Structural and bacterial transitions from flocs to granules in the bubble-column SBR

**Figure 4:**
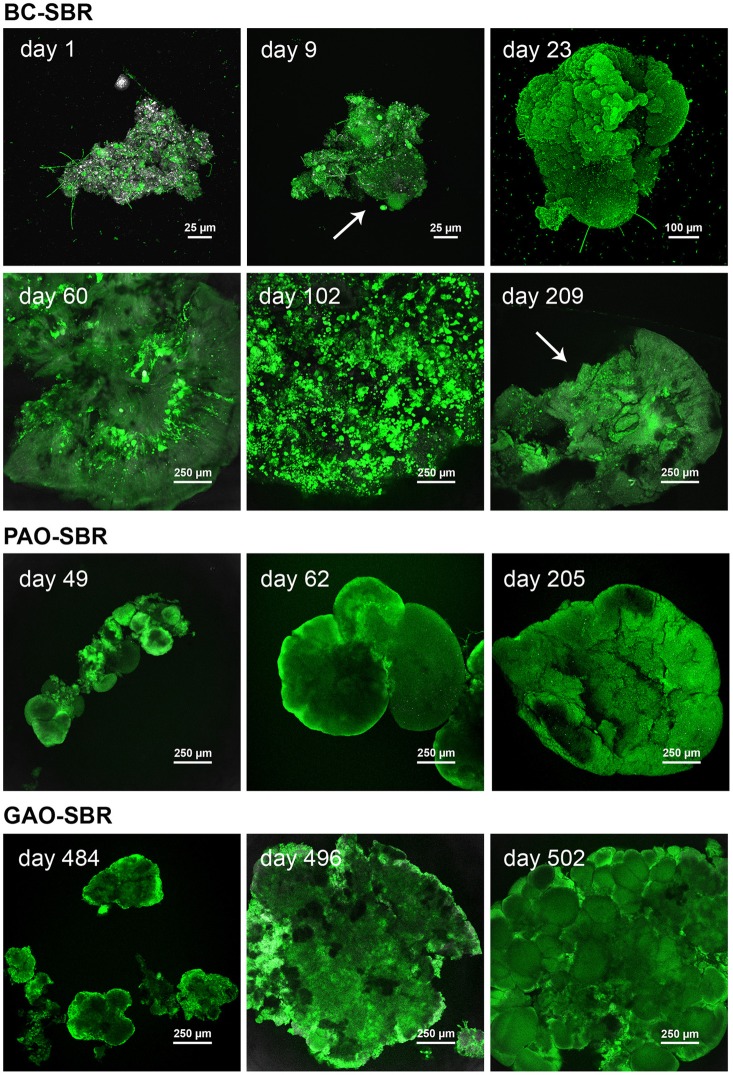
**Temporal evolution of the architecture of bioaggregates from activated sludge flocs to early-stage granules and mature granular biofilms in the BC-SBR, PAO-SBR, and GAO-SBR**. CLSM datasets were recorded on full bioaggregates from samples taken up to day 23, 62, and 484 in the BC-SBR, PAO-SBR, and GAO-SBR, respectively. Granules from later samples were analyzed on cross-sections. The sample taken on day 209 in the BC-SBR was analyzed as 80-μm cryosection. The green fluorescent dye Rhodamine 6G was used to map cells and biofilm matrices. In 8 bit data sets, 256 green levels were allocated to this dye. The reflection signal was used as reference with 256 gray/white color allocation. In the BC-SBR, swelling of microbial colonies around the floc structure can be observed on day 9. Early-stage granule nuclei on day 23 were 4–5 times bigger than flocs, and displayed compact biofilm aggregation. The evolution of the internal architecture of granules with proliferation of dense microcolonies from the granule core outwards can be observed from CLSM images taken on day 60 and 102. After more than 200 days, mature granules displayed a heterogeneous internal structure with internal voids and detachment. Granules formed in the PAO-SBR by proliferation of dense microbial clusters around the floc structure (day 49). Dense granule nuclei obtained after 62 days evolved toward mature granules exhibiting folded biofilm structures (day 205). In the GAO-SBR dense nuclei (day 484) evolved in 2 weeks toward 4–5 times larger granular biofilms (day 496). On day 510, granules were characterized as heterogeneous conglomerates of dense microbial clusters.

CLSM examinations of structural dynamics of bioaggregates in the BC-SBR with Rhodamine 6G staining are presented in Figure 4 and Supplementary material 6. Amorphous flocs (150–200 μm) initially present in the BC-SBR (day 1) underwent transformation by swelling of microbial colonies around flocs (day 9), followed by granulation of nuclei with dense rounded structures of 450–750 μm (day 23). Early-stage granules (850–1500 μm) displayed smooth and folded biofilm structures (day 30). Between days 50 and 140, round and compact microcolonies (10–100 μm) followed by larger ones (120–300 μm) proliferated from the inner core of granules outwards. Granular biofilm detachment was detected on day 60, and increased during maturation (day 112). Mature granules comprised internal voids, large biofilm clusters, and slimy interfacial matrices (days 209 and 218).

**Figure 5:**
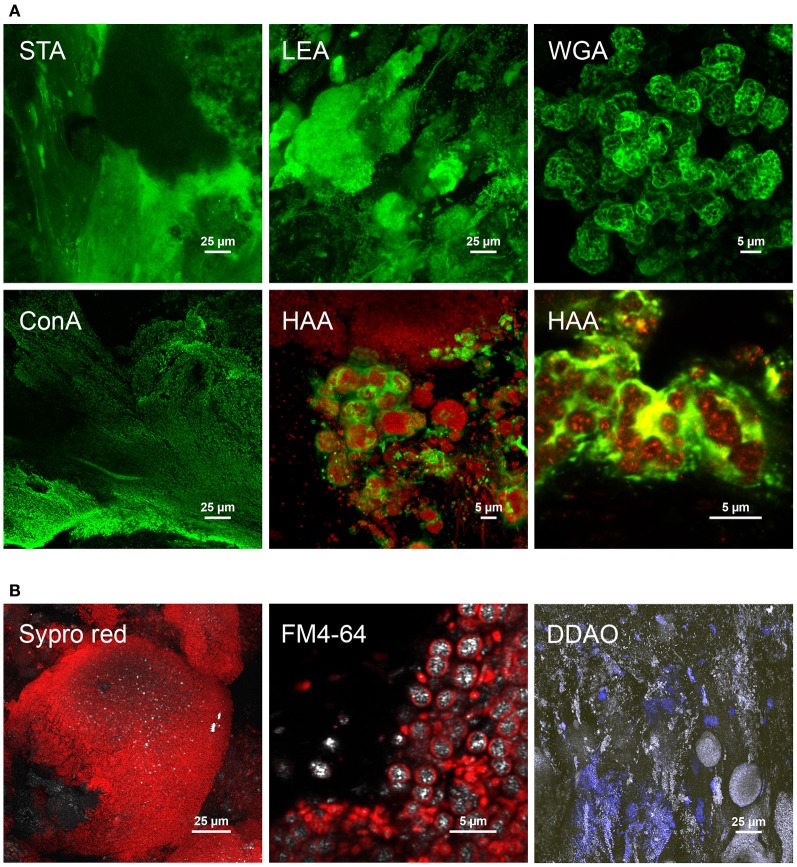
**Examples of cellular and extracellular features detected in cross-sectioned granular biofilms collected after 85 days in the BC-SBR. (A)** Selected glycoconjugate signals by means of FLBA. The STA lectin revealed the presence of a wide glycoconjugate matrix across the granule sections. Biofilm growth directions from the inner core to the outer sphere can be observed on the left part of the image with the growth lines displayed with the lectin staining. LEA showed dense microcolonies surrounded by a glycoconjugate matrix. WGA clearly showed glycoconjugate matrices surrounding specific types of microcolonies. ConA stained cell surface glycoconjugates also indicating biofilm growth lines and directions. HAA was used in combination with SYTO 60 and FM4-64 fluorescent probes. Color allocations: lectins binding to glycoconjugates (green), cell staining (red). **(B)** Further structures detected with additional fluorescent probes in the architecture of mature granules collected after 111 days in the BC-SBR. Dense spherical microbial clusters stained with SYPRO Red. Detection of microbial cells comprising bright reflecting intracellular storage compounds (cell membranes were stained with FM4-64). Presence of extracellular DNA stained with DDAO in the biofilm matrix and around cell clusters. Color allocations: fluorescent probes (red), reflection signal (gray).

Different glycoconjugates were detected by fluorescent lectin-binding analysis (FLBA) of cross-sections of granules collected on days 85 and 105. According to CLSM data provided in Figure 5A and in Table SM3.2 in Supplementary material 3, FLBA showed (1) embedded continuous matrices revealed by STA, PHA-L, and IAA lectins, (2) matrices surrounding larger microbial clusters (HAA, LEA, SBA), (3) matrices of microcolony interfaces (WGA, LcH), and (4) direct binding to cell surfaces (ConA). HAA also revealed filamentous sheaths, and ConA the outwards palisade-like orientation of the biofilm continuum that surrounded dense colony clusters. Further interesting structures were detected in the architecture of mature granules with the use of other fluorescent probes (Figure 5B), e.g., spherical dense colony clusters of about 140 μm stained with SYPRO Red, aggregation of cells containing bright reflecting intracellular storage compounds after staining membranes with FM4-64, and extracellular DNA stained with DDAO.

**Figure 6:**
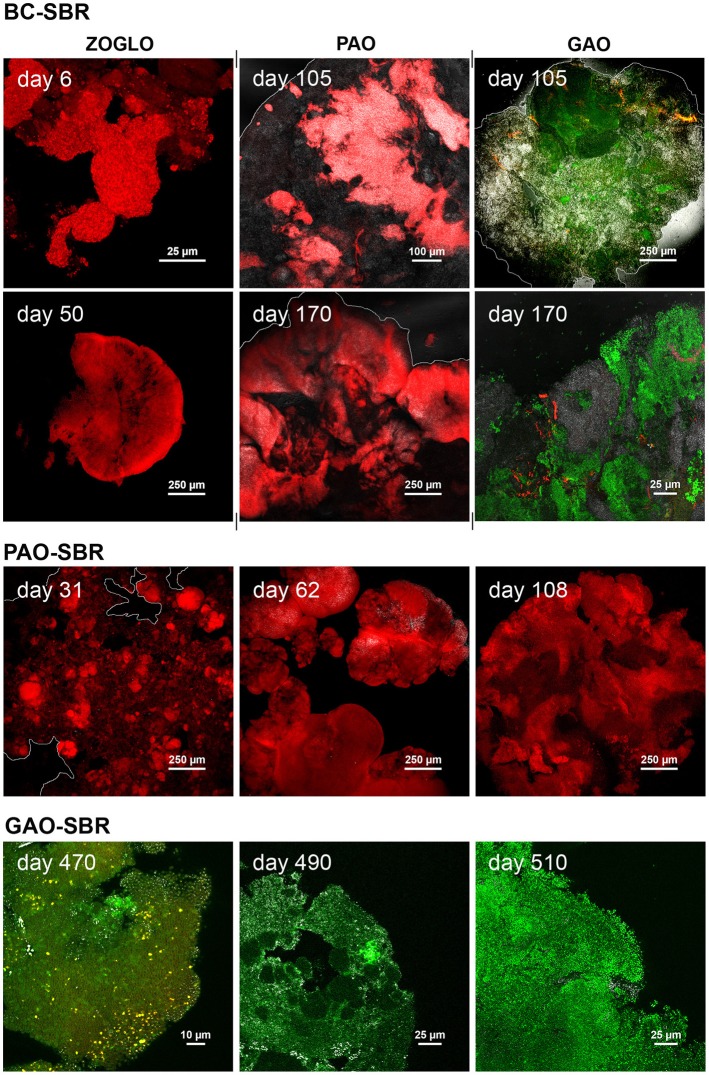
**FISH-CLSM analyses of spatial dynamics of target bacterial populations during granule formation and maturation in the BC-SBR, PAO-SBR, and GAO-SBR**. In the BC-SBR, colonies of *Zoogloea* (ZOGLO gene probe, red) swole around the floc structure on day 6 and formed the smooth biofilm continuum of early-stage granules on day 50. Biofilm growth lines from granule core outwards can be observed on this image. After more than 100 days, *Accumulibacter* (PAO, red) and *Competibacter* (GAO, green) proliferated inside mature granules from inner core outwards and were predominant in mature granules analyzed on day 170. On days 105 and 170, *Zoogloea* (shown in red in the GAO-related images) were only detected in low abundances in interstices between the different other microbial clusters. In the PAO-SBR, active *Accumulibacter* affiliates (red) predominated during granule formation. This population formed the dense bacterial clusters that proliferated in the floc structure (day 31), and that led to the formation of heterogeneous dense granules composed of bright reflecting clusters (day 62). The image of day 108 exhibits *Accumulibacter* coverage over the whole granule cross-section. In the GAO-SBR, *Competibacter* (GAO, green) was predominant during granulation. On day 470, the microbial cell membranes were stained with the fluorescent probe FM4-64 (red/yellow) after hybridization with the GAO gene probe. On day 490, the granules were composed of different GAO populations present in a continuous matrice and in dense microbial clusters, respectively. On day 510, granules displayed predominance of GAO populations with round cells.

FISH-CLSM confirmed T-RFLP analyses and provided information on spatial dynamics of active bacteria inside the structure of bioaggregates (Figure 6 and Supplementary material 7). *Zoogloea* proliferated during granulation as microcolonies of 20–45 μm swelling around flocs (day 6), and formed the early-stage granular biofilm continuum (day 50). It can be observed on the latter image that early-stage granules displayed loose core and dense surface aggregation. *Zoogloea* disappeared from the biofilm architecture of mature granules, as displayed on day 170. PAO and GAO were present as microcolonies (<10 μm) in the flocculent sludge. After 100 days, PAO and GAO established over *Zoogloea* from the granule cores outwards by forming large and dense clusters (>300 μm) exhibiting bright and low reflection, respectively. After initial presence in flocs as compact microcolonies of up to 30 μm (day 6), AOO proliferated as dense microcolonies (20–70 μm) across granules after 105 days and into wider biofilm matrices near the granule surface after 170 days.

### Granulation in the stirred-tank PAO-SBR and GAO-SBR

According to Figure 4 and Supplementary material 8, granulation occurred in the PAO-SBR successively (1) by floc smothering (day 30), (2) by proliferation of round and dense clusters of 90–180 μm in flocs (day 49), and (3) by formation of smooth and dense nuclei that evolved up to 1.3-mm early-stage granules (day 62) and 1.5–2.0 mm mature granules (day 205). Mature granules displayed folded biofilm structures that contained aggregation of cells in dense biofilm clusters (day 205 and 215).

FISH-CLSM measurements (Figure 6) confirmed that PAO dominated during granulation by forming small dense clusters (10–120 μm) in smooth globular flocs (day 31), and larger dense clusters (<500 μm) in early-stage granules (day 62). PAO occupied the entire cross-sections of mature granules after 108 days. PAO cells (1.5 μm) had a high content of bright-reflecting intracellular storage compounds, and were more densely aggregated over the first 200–300 μm from the granule surface. *Zoogloea* were almost absent during granulation in the PAO-SBR, by forming only low abundant colonies (10–20 μm) in flocs (day 5) and patches at the surface of granule nuclei (day 31) (Supplementary material 7). *Zoogloea* were only present in biofilm interstices after 62 days.

In the GAO-SBR, flocs turned into compact nuclei of 200–600 μm after 484 days that evolved up to larger 1.3–1.5 mm granules (day 496) (Figure 4). Granules present in the GAO-SBR were heterogeneous conglomerates of large and dense clusters of 50–350 μm comprising 2.0–2.5 μm sized cells (day 510). FISH-CLSM analyses revealed that granulation occurred in this reactor under predominance of GAO across granules (Figure 6).

### Correlation between granule structures and predominant populations

**Figure 7:**
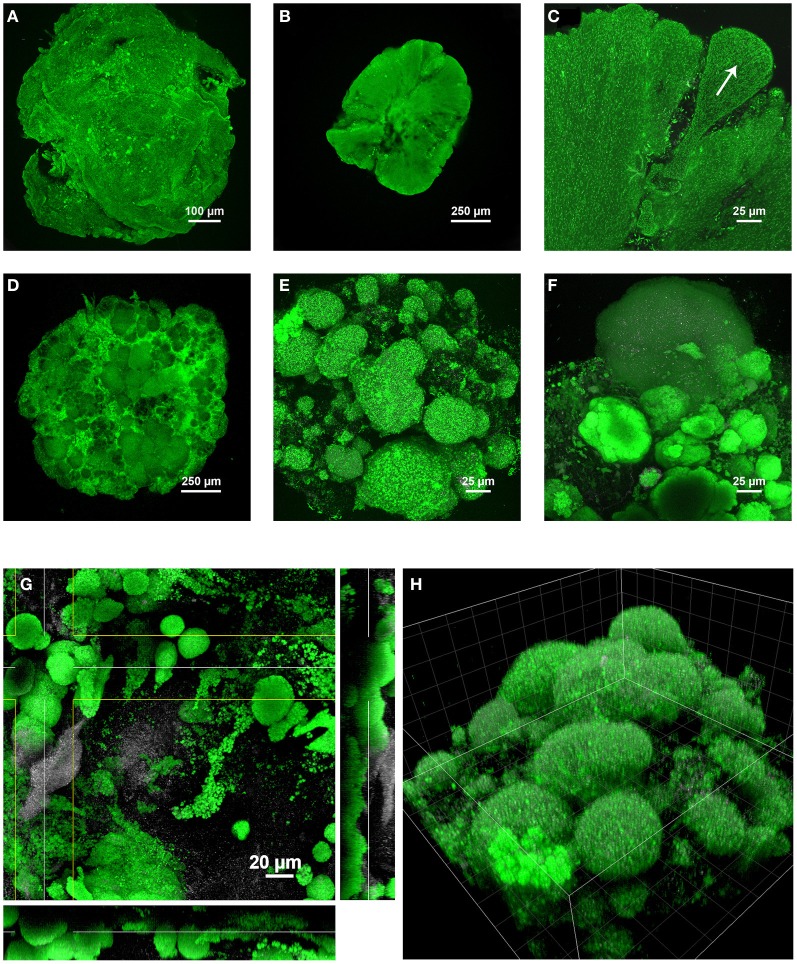
**Main differences in the architectures of granules depending on the predominant bacterial populations involved, examined in two (A–F) and three dimensions (G,H)**. Under predominance of *Zoogloea* spp., smooth granular biofilm continua were obtained in early-stage granules collected after 20 days **(A)** and 60 days **(B)** in the BC-SBR. The formation and the outgrowth direction of biofilm petals can be observed in **(C)**. The biofilm was composed of a homogeneous cell distribution in a gel matrix. Under conditions favoring PAO and GAO growth heterogeneous granular biofilm conglomerates were obtained as exemplified by day 503 **(D)** and day 484 **(E)** in the GAO-SBR, and by day 140 in the BC-SBR **(F)**. During the transition on day 80 from early-stage to mature granules in the BC-SBR, the granular biofilm was composed of heterogeneous microbial clusters proliferating from the granule core outwards **(G**, XYZ projection**)**. The cauliflower-like structure of heterogeneous granules present in the GAO-SBR is comparable to the mushroom-like structures of biofilm growth on a solid substratum under substrate-limiting conditions **(H**, 3-D volume projection, grid size of 20 μm**)**. Color allocations: Rhodamine 6G (green), reflection signal (gray).

Based on the different structural time series described above for the three SBRs, the structure of granules correlated with the predominant organisms involved. A compilation of main structural differences is provided in Figure 7. Over the first 60 days in the BC-SBR, *Zoogloea*-dominated granules displayed smooth external and internal granular biofilm architectures (Figures 7A,B). In this case, granules displayed homogeneous embedment of cells in a biofilm matrix growing outwards like petals (Figure 7C). Granules dominated by slower-growing PAO and GAO displayed heterogeneous aggregation of population clusters (Figures 7D–F). In the GAO-SBR, cauliflower-like structures were observed from the surface of granules (Figure 7E).

Additional features of granules are presented in Supplementary material 9. Different cell distributions were observed in cross-sections of early-stage granules collected on day 50 (A) and of mature granules collected on day 111 in the BC-SBR (B). Mature granules dominated by PAO in the BC-SBR displayed heterogeneous internal architectures closer to the ones of granules cultivated in the PAO-SBR (C). Further specific structures are the initial microcolonies that swelled in flocs after 6 days in the BC-SBR (D), the filamentous populations that were detected after 11 days in the flocs of the PAO-SBR (E), and the slimy matrices present at the surface of mature granules after 209 days in the BC-SBR (F).

### Three-dimensional analyses of granule structures

Specific biofilm and microbial structures examined by CLSM were finally analyzed in three dimensions (3-D) either as XYZ projections or volumetric presentations. During the transition from early-stage to mature granules after 80 days, the internal architecture of granules was composed of various types of microcolonies such as dense spherical microbial clusters proliferating from the granule core and populations aggregated in looser amorphous structures (Figure 7G). The volumetric presentation of the granular aggregates obtained in the GAO-SBR on day 484 exhibit the cauliflower-like or mushroom-like cluster evolving from the granule core outwards (Figure 7H).

Additional 3-D examinations are provided in Supplementary material 10. The early-stage granule obtained after 20 days in the BC-SBR displayed surface roughness with biofilm protuberances and valleys (A). After 111 days, a zoom on a microcolony revealed a spherical shape and denser cell aggregation at the edge of the microcolony (B). On the same day, dual staining with SYTOX Green (nucleic acids label) and Nile Red (label for hydrophobic components such as poly-β-hydroxyalcanoates) enabled to detect different types of cells based on their membrane and intracellular properties (C). Other volumetric presentations display the 3-D orientation of the glycoconjugate matrices detected with the WGA lectin around microcolony interfaces (D) and the finger-like population structures detected in early-stage nuclei obtained after 15 days in the BC-SBR that was dominated by *Zoogloea* (E).

## Discussion

### Granulation can occur under wash-out and steady-state conditions

Granulation of activated sludge occurred under wash-out and steady-state conditions with different SBR designs. Wash-out conditions in the BC-SBR resulted in fast granulation over 2 weeks, which agrees with the granule formation periods reported by Beun et al. (1999). Mosquera-Corral et al. (2011) have succeeded to cultivate granules in a fully aerated stirred-tank SBR by imposing wash-out with short settling time (1 min) and HRT (6 h) right from start-up. In the present study, spontaneous granulation occurred in the stirred-tank SBRs run at steady-state to cultivate PAO and GAO enrichments. This phenomenon has also been observed in various stirred-tank SBRs operated for BNR (Dangcong et al., 1999; de Villiers and Pretorius, 2001; Dulekgurgen et al., 2003; You et al., 2008; Barr et al., 2010). Granulation in the GAO-SBR correlated with over-aeration that could have induced a physiological stress by increased endogenous respiration processes. Although three times slower than with wash-out, spontaneous granulation occurred in the PAO-SBR operated at steady-state under conditions selecting for *Accumulibacter*, with full control of the anaerobic uptake of VFA by step-wise adaptation of the OLR and anaerobic contact time, with stable SRT between 8 and 10 days, and with not very short settling time (60–10 min), and HRT (12 h). Fast-settling nuclei formed despite initially long 60-min settling periods. Such enhancement of biomass settling can be explained by the fact that dephosphatating sludge is denser than conventional activated sludge because of the presence of organic and inorganic intracellular polymers inside PAO cells (Schuler et al., 2001). The decrease of settling time from 60 to 10 min probably further selected for faster settling aggregates and full granulation. Hence, although wash-out conditions are efficient for fast granulation, granulation can also occur spontaneously. The granulation phenomenon in stirred-tank SBRs should further be investigated in a statistical approach in order to identify the trigger factors of granulation in this particular type of reactors. In addition, wash-out conditions can lead to prolonged deteriorated BNR since proper conditions to select for active PAO and nitrifiers are often not fulfilled (Weissbrodt et al., 2012a). Intermediate conditions between wash-out and steady-state should therefore be investigated for rapid formation of granules with good BNR activities.

### Bacterial ecology considerations

Since *Tetrasphaera* have been reported as important phosphorus-removing and glucose-fermenting organisms of full scale BNR-WWTP (Nielsen et al., 2012a,b), their regular presence in AGS-SBRs can be explained by the alternation of anaerobic-aerobic conditions and the high content of exopolysaccharides present in granules. The longest persistence of *Tetrasphaera* in the GAO-SBR at high abundances revealed that this population can cope with operation at higher mesophilic temperature and slight acidic pH, what could be relevant for dephosphatation of warm and acidic wastewaters. The GAO-enrichment culture revealed abundant *Rhodospirillaceae*-related *Alphaproteobacteria*. *Defluviicoccus vanus* which belongs to *Rhodospirillaceae* has been reported as a putative alphaproteobacterial GAO (Meyer et al., 2006), that is selected for with propionate as substrate (Lopez-Vazquez et al., 2009b). Here, *Rhodospirillaceae* relatives were abundant despite the presence of only acetate as substrate.

Nitrogen removal in the BC-SBR correlated with dynamics of denitrifiers. Although clades of *Accumulibacter* (and *Competibacter*) can denitrify and are desired for denitrifying dephosphatation (Yilmaz et al., 2008; Oehmen et al., 2010), denitrification in anaerobic-aerobic AGS-SBRs is not restricted to only PAO and GAO. Other denitrifiers that presumably do not take up VFA anaerobically were present in the full anaerobic-aerobic AGS ecosystems. Denitrifying metabolic activities and utilization of exopolysaccharides as electron donors in AGS systems should be investigated further based on previous knowledge gained from activated sludge systems (Finkmann et al., 2000; Thomsen et al., 2007; Ni et al., 2009; Nielsen et al., 2012b).

**Figure 8:**
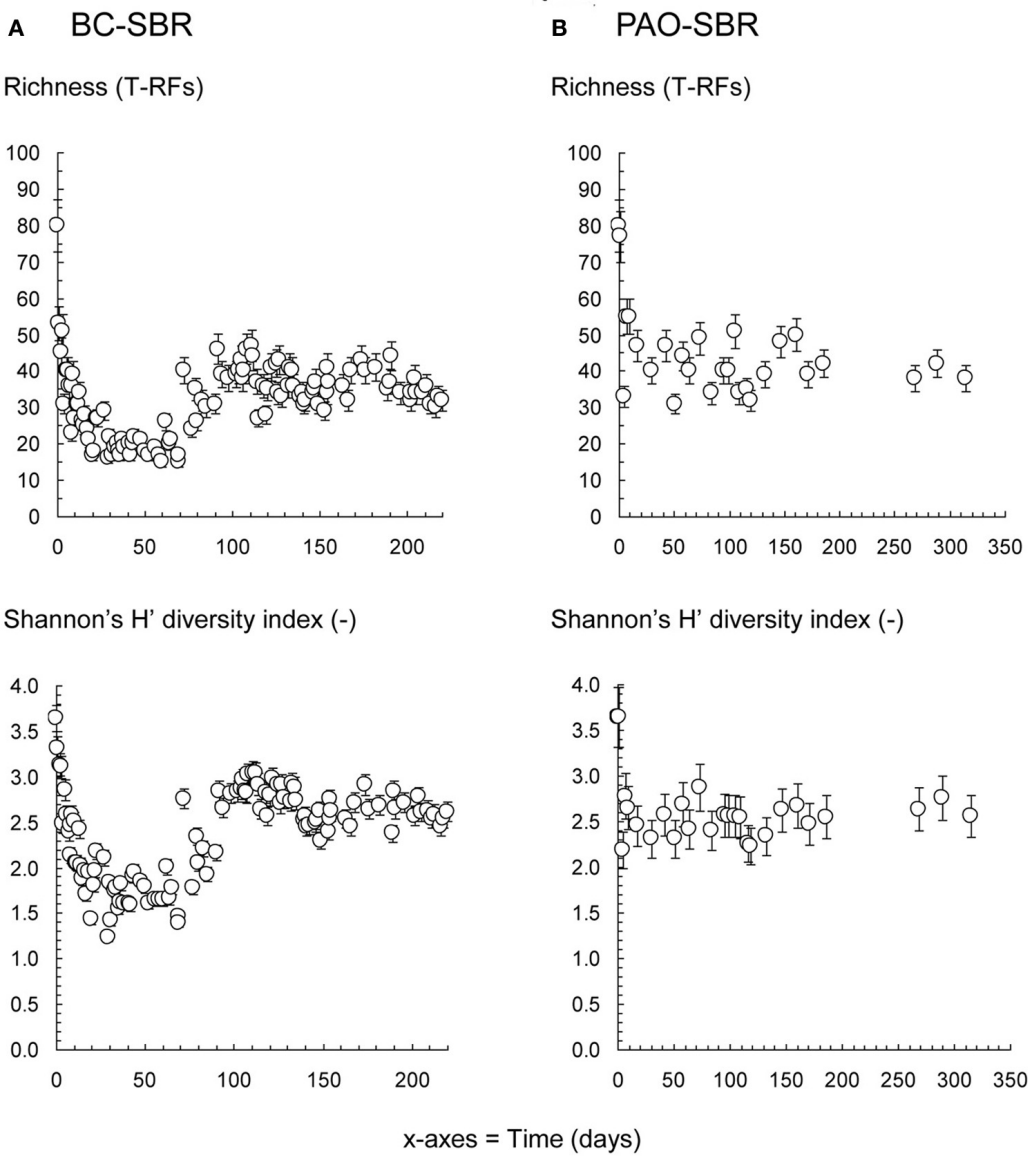
**Evolutions of the richness and Shannon's H' diversity indices of the bacterial communities present in the BC-SBR (A) and PAO-SBR (B) computed based on the measured T-RFLP profiles**. The application of wash-out conditions in the BC-SBR impacted on both indices over the first 70 days. A richer and more diverse community re-established after more than 80 days under the process conditions that impaired the *Zoogloea* proliferation. The operation of the PAO-SBR under steady-state conditions by full control of operation conditions resulted in stable richness and diversity of the underlying community. In both SBRs, initial decreases in richness from 80 to <50 T-RFs and in diversity from 3.7 to <3.0 were observed over the first 5 days after inoculation with the activated sludge from the full-scale plant and resulted from reactor feeding with VFA-based synthetic wastewaters.

The richness and diversity patterns, which are commonly used to characterize biological and wastewater systems (Liu et al., 1997; Borcard et al., 2011; Gonzalez-Gil and Holliger, 2011; Winkler et al., 2012), were used to compare the evolution of the overall bacterial community structure under wash-out and steady-state conditions (Figure 8). In addition to the drop in richness and diversity during early-stage granulation reported in Weissbrodt et al. (2012a), wash-out conditions more intensively impacted on these indices. Under steady-state conditions in the PAO-SBR, granulation occurred with stable and relatively high community indices, despite first decrease due to synthetic laboratory conditions in the very beginning.

### Granulation mechanisms depend on process conditions and predominant organisms

Granulation occurred under predominance of *Zoogloea* (BC-SBR), *Accumulibacter* (PAO-SBR), as well as *Competibacter* and *Sphingobacteriales* relatives (GAO-SBR). Thus, *Zoogloea* seem not to be essential for granule formation, answering an open question from earlier work where *Zoogloea* was predominant in dense fast-settling granules in different start-up experiments of AGS BC-SBRs (Weissbrodt et al., 2012a). According to Hesselsoe et al. (2009) and Nielsen et al. (2010), the predominant organisms that were observed in granules share physiological functions required for biofilm formation, namely production of exopolysaccharides and surface adhesins.

Whereas *Zoogloea*, *Xanthomonadaceae*, *Sphingomonadales*, and *Rhizobiales* relatives can contribute to the production of e.g., zooglan, xanthan, and sphingan exopolysaccharides (Lee et al., 1997; Pollock et al., 1998; Denner et al., 2001; Dow et al., 2003), *Sphingobacteriales* affiliates consume exopolysaccharides (Matsuyama et al., 2008). Granules can therefore be considered as exopolysaccharide-based ecosystems of bacterial producers and consumers. Seviour et al. (2011, 2012) have isolated a specific exopolysaccharide (granulan) from GAO-dominated granules, whereas Lin et al. (2008, 2010) have highlighted alginate as key granular exopolymer. The here applied *in situ* assessment of specific glycoconjugates by FLBA can inform on the type of abundant sugar residues (Zippel and Neu, 2011) present in the granules examined. N-acetyl-glucosamine (GlcNAc) multimers were observed in the biofilm continuum (STA lectin) and monomers in matrices surrounding microcolonies (LEA, WGA), N-acetyl-galactosamine (GalNAc) around bacterial clusters (HAA, SBA), and α-glucose or α-mannose at (*Zoogloea*-) cell surfaces dispersed in the continuum (ConA). Whereas the GAO-related granulan heteropolysaccharide isolated by Seviour et al. (2012) comprises GalNAc residues, the exopolysaccharides produced by *Zoogloea ramigera* contain glucose and galactose. The diversity of polymer matrices detected here indicated, however, that granules are composed of a complex mixture of glycoconjugates and that there is probably not only one key exopolymer present in all aerobic granules. Since exopolysaccharide presence depends on predominant microorganisms involved and on specific growth and operation conditions (Nielsen et al., 2004), functional screening should operate on a systems microbiology approach. Glycoconjugates and bacterial populations could be co-localized by combined lectin-binding and FISH analyses (Böckelmann et al., 2002). The microbial ecology data collected in this study indicated that only a low number of microorganisms would have to be screened in BNR AGS systems.

Different granulation mechanisms were observed depending on process conditions and predominant organisms involved. Predominance of fast-growing *Zoogloea* under non-limiting substrate conditions resulted in homogeneous granular biofilm matrices of cells dispersed in a gel. This biofilm continuum was formed by microcolony swelling around flocs, and embedded further proliferation of dense clusters of slower-growing nitrifiers, PAO, and GAO. This picture is similar to experimental and model-based descriptions of multispecies biofilms and underlying cooperative and non-cooperative microbial interactions (Picioreanu et al., 2000, 2004; Alpkvist et al., 2006; Alpkvist and Klapper, 2007; Xavier and Foster, 2007). Fast-growing heterotrophic competitors apparently formed the embedding biofilm continuum by production of exopolysaccharides and rapid proliferation outwards. Biofilm growth against substrate gradients explains the palisade-like orientation of cell lines, which has also been shown in methanogenic granules (Batstone et al., 2006). This converges to the first hypothesis of Barr et al. (2010) on the granulation mechanism by microcolony outgrowth, and on the early statement of Characklis (1973) that attached “*microbial growth originates in a mixture of slime and zoogloeal bacteria (microorganisms that form gelatinous aggregates)*.”

Proliferation of nitrifiers, PAO, and GAO as dense clusters transported by the zoogloeal matrix may rely on the slower growth rates exhibited by these populations compared to fast-growing heterotrophic ones (de Kreuk and van Loosdrecht, 2004; Okabe et al., 2004). Accumulation of *Accumulibacter* resulted in denser granular biofilms, something which can explain, in addition to higher polyphosphate contents, the slight decrease in bed height in the BC-SBR despite increase of the biomass concentration between days 90 and 110. At mature stage in all reactors, the *Accumulibacter*- and *Competibacter*-dominated granules displayed heterogeneous aggregation of dense bacterial clusters in a cauliflower-like structure. Formation of heterogeneous biofilms has also been related to growth rate considerations under substrate limitations (Picioreanu et al., 2000; Alpkvist et al., 2006) that occurred as soon as full anaerobic feast and aerobic growth under starvation were achieved. Whereas heterogeneous architectures of mature granules can be explained by microcolony re-aggregation after detachment (Barr et al., 2010). Differences in bacterial physiologies can also lead to formation of different granular shapes by proliferation in single granules of either fast-growing organisms in a smooth continuum or slower-growing ones in heterogeneous dense clusters.

Biofilm growth is limited by detachment (Morgenroth, 2008). Detachment not only occurred at granule surfaces, but on entire biofilm whorls starting from the core of granules. Such detachment can result in dual access of substrates by the surface and the core of granules, what is comparable to biofilms growing on porous membranes with substrate penetrating from both sides (Downing and Nerenberg, 2008), and should be considered in mass transport phenomena across granules. The FISH-CLSM micrographs suggested that the proliferation of *Accumulibacter*, *Competibacter*, and nitrifiers clusters occurred from granule core outwards. This might be sustained by detachment phenomena and the access of substrates to central granule zones. Similarly to planar biofilms and to what has also been observed by different authors (Lemaire et al., 2008a; Lee et al., 2009; Barr et al., 2010), granules exhibit more complex structures than stratified architectures considered in conceptual and mathematical models. Granules heterogeneities can also explain the differences in the spatial organization of target organisms in the granular biofilm ecosystems depending on the process operation.

In conclusion, the knowledge gained on the complex bacterial and structural dynamics during granule formation and maturation led to the following findings. *Zoogloea*, *Accumulibacter*, and *Competibacter* can form granules and therefore *Zoogloea* are not essential for granulation. Granulation mechanisms depend on operation conditions and predominant organisms involved. *Zoogloea* form homogenous biofilms embedding the development of nitrifiers, PAO, and GAO colonies. *Accumulibacter* and *Competibacter* form heterogeneous aggregates of dense clusters. Mature granules display complex internal architectures exhibiting interspersing channels and biofilm detachment that can favor substrate access and growth of bacterial clusters from granule core outwards. Granulation of active *Accumulibacter* populations was possible under steady-state conditions under full control of the OLR and anaerobic contact time. Under such conditions, granulation of activated sludge was, however, three times slower than under wash-out conditions. Further studies targeting rapid formation of actively dephosphatating granules from flocculent sludge should therefore find a compromise between wash-out and steady-state conditions. The present fundamental study was performed with VFA-based synthetic wastewater. As additional research objective, one may investigate the impact of real wastewater compositions and particulate substrates on granulation mechanisms.

### Conflict of interest statement

The authors declare that the research was conducted in the absence of any commercial or financial relationships that could be construed as a potential conflict of interest.

## Supplementary material

The SupplementaryMaterial for this article can be found online at: http://www.frontiersin.org/Microbiotechnology,_Ecotoxicology_and_Bioremediation/10.3389/fmicb.2013.00175/abstract

Table SM1.1 **Composition of the synthetic influent wastewaters fed in the BC-SBR, and in the stirred-tank PAO-SBR and GAO-SBR**.

Table SM1.2 **Composition of trace element solutions**.

Figure SM2.1 **Schemes of the bubble-column and stirred-tank SBRs**.

Table SM3.1 **Efficiency of fluorescent dyes and of glycoconjugate-binding lectins for structural analysis of flocs and granules with CLSM**. Efficiency of fluorescent dyes for investigating flocs and granules structures by CLSM.

Table SM3.2 **Efficiency of lectins for targeting glycoconjugates in the matrices of granular biofilms by means of CLSM**.

Table SM4.1 **Targeted 16S rRNA oligonucleotide probes used to follow by FISH-CLSM the temporal and spatial evolutions of *Zoogloea*, *Accumulibacter*, *Competibacter*, and ammonium-oxidizing organisms (AOO) during granule formation**.

Table SM5.1 **Closest phylogenetic bacterial affiliations of OTUs detected in the BC-SBR, that were obtained with the pyrosequencing-based bioinformatics PyroTRF-ID**.

Table SM5.2 **Closest phylogenetic bacterial affiliations of OTUs detected in the PAO-SBR obtained with PyroTRF-ID**.

Table SM5.3 **Closest phylogenetic bacterial affiliations of OTUs detected in the GAO-SBR obtained with PyroTRF-ID**.

Figure SM6.1 **Structural dynamics during granule formation and maturation in the BC-SBR**. Temporal evolution of the architecture of bioaggregates from activated sludge flocs (day 1) to early-stage (day 23) and mature granular biofilms (day 102) in the BC-SBR operated under wash-out conditions. CLSM datasets were recorded on full bioaggregates from samples taken from day 1 to 23, as well as on day 218. Granules from samples taken between day 30–140 were analyzed on cross-sections. The sample taken on day 209 was analyzed as 80-μm cryosection. The green fluorescent dye Rhodamine 6G was used to map cells and biofilm matrices. In 8 bit data sets, 256 green levels were allocated to this dye. The reflection signal was used as reference with 256 gray/white color allocation. On day 9, swelling of microbial colonies around the floc structure can be observed. Early-stage granule nuclei on day 23 were 4–5 times bigger than flocs, and displayed compact biofilm aggregation. On day 30, early-stage granules were composed of a continuous biofilm displaying homogenous cell distribution and folded structures. From day 30 to 102, the internal architecture of granules evolved with the proliferation of dense microcolonies from the granule core outwards. Larger microbial clusters appeared in the structure of granules between day 112 and 140. Detachment phenomena contributed to the heterogeneous structure of mature granules (days 112 and 209). After more than 200 days, mature granules exhibited aggregation of dense biofilm clusters, internal voids, and eroded surface slimy structures.

Figure SM7.1 **Spatial dynamics of additional bacterial populations during granule formation and maturation in the BC-SBR and PAO-SBR**. The FISH-CLSM analyses revealed that *Accumulibacter* affiliates (PAO, red) were initially present in the flocculent activated sludge on day 6 in the BC-SBR. These organisms and their competitors affiliating with *Competibacter* (GAO, green) were only present in low abundances on day 50, and were outcompeted by *Zoogloea* spp. (Figure 6). Ammonium-oxidizing organisms (AOO, green) were detected on day 6 inside flocs. During AGS maturation, AOO proliferated across granules as dense microcolony clusters (day 105), and form wider population matrices near edges of mature granules (day 170). In the PAO-SBR, *Zoogloea* spp. (ZOGLO, red) were never abundant during granulation in the PAO-SBR.

Figure SM8.1 **Temporal evolution of the architecture of bioaggregates from flocs to early-stage granules up to mature granules in the stirred-tank PAO-SBR operated under steady-state conditions**. Granule formation occurred in the PAO-SBR by smothering of flocs (day 30) and proliferation of dense microbial clusters around the floc structure (day 49). Dense granule nuclei obtained after 62 days evolved toward mature granules exhibiting folded biofilm structures (day 205) and aggregation of big, dense and homogeneous clusters (day 215).

Figure SM9.1 **Additional structural features of bioaggregates detected during granulation**. The cross-section of an early-stage granule collected on day 50 in the BC-SBR and hybridized with *Zoogloea*-targeted gene probes **(A)** revealed a more homogeneous cell composition than the highly-heterogeneous “bacterial metropolis” of a mature granule cross-section collected on day 111 in the same reactor and stained with SYPRO Red **(B)**. **(A)** also provides information on the palisade-like biofilm outgrowth lines, and on the denser cell aggregation on the first 75 μm from the granule edge. The heterogeneous structure of mature granules obtained in the BC-SBR, that is actually dominated by PAO on day 111, is similar to the one of granules cultivated in the PAO-SBR after 31 days **(C**, color allocation: red, PAO gene probe**)**. Further structures were detected by staining with Rhodamine 6G, such as the globular microcolonies swelling around flocs after 6 days in the BC-SBR **(D)**, filamentous populations detected in the bioaggregates of the PAO-SBR after day 11 **(E**), and slimy structures at the surface of mature granules collected after 209 days in the BC-SBR **(F)**.

Figure SM10.1 **Three-dimensional examinations of specific structures of granular biofilms**. The 3-D analyzes were conducted with XYZ projections **(A–C)** and 3-D volume projection **(D,E)**. The smooth early-stage granular biofilms on day 20 in the BC-SBR exhibited a surface roughness with biofilm protuberances and valleys **(A)**. A zoom on a spherical microcolony in a mature granule on day 111 in the BC-SBR revealed denser cell aggregation at the edge of the colony **(B)**. Dual staining with SYTOX Green (nucleic acids) and Nile Red (hydrophobic cellular components and poly-β-hydroxyalcanoates) revealed different cell types in the heterogeneous structure of mature granular biofilms on day 111 in the BC-SBR **(C)**. The WGA lectin highlights the 3-D distribution of glycoconjugate matrices surrounding interfaces of microcolony clusters **(D**, grid size of 5 μm**)**. Finger-like zoogloeal population structures were detected in the architecture of aggregates collected on day 15 in the BC-SBR **(E**, grid size of 5 μm**)**. Color allocation in **(A**,**B**, and **E)**: Rhodamine 6G (green) and reflection (gray/white).
