# Supplementary material for: Assessment of bacterial and structural dynamics in aerobic granular biofilms
Source: Front Microbiol. 2013 Jul 10;4:175. doi: 10.3389/fmicb.2013.00175 (PMC3707108; doi:10.3389/fmicb.2013.00175)
Supplement: Table SM1.1 — Composition of the synthetic influent wastewaters fed in the BC-SBR, and in the stirred-tank PAO-SBR and GAO-SBR. [file DataSheet1.PDF]

Assessment of bacterial and structural dynamics in aerobic granular biofilms

SUPPLEMENTARY MATERIAL

David G. Weissbrodt[1,*], Thomas R. Neu[2], Ute Kuhlicke[2], Yoan Rappaz[1], Christof Holliger[1]

[1] Laboratory for Environmental Biotechnology, School for Architecture, Civil and Environmental Engineering, Ecole Polytechnique Fédérale de Lausanne, Lausanne, Switzerland

[2] Microbiology of Interfaces, Department of River Ecology, Helmholtz Centre for Environmental Research - UFZ, Magdeburg, Germany

*Correspondence:
Dr. David Weissbrodt
EPFL ENAC IIE LBE
Station 6
CH-1015 Lausanne, Switzerland
e-mail: david.weissbrodt@epfl.ch

E-mail addresses: DGW – david.weissbrodt@epfl.ch ; TRN – thomas.neu@ufz.de ; UK – ute.kuhlicke@ufz.de ; YR – yoan.rappaz@epfl.ch ; CH – christof.holliger@epfl.ch

Supplementary material 1

Table SM1.1 Composition of the synthetic influent wastewaters fed in the BC-SBR, and in the stirred-tank PAO-SBR and GAO-SBR.

Compound	CAS no.	Molecular formula	Molecular weight (g mol^{-1})	Concentration in influent wastewater		
				BC-SBR[2]	PAO-SBR[3]	GAO-SBR[3]
**C-source medium**[1]					**(mmol L^{-1})**	
Sodium acetate[4]	127-09-3	$C_2H_3O_2Na \cdot 3H_2O$	136.09	7.81	-	6.25
Sodium propionate[4]	137-40-6	$C_3H_5O_2Na$	96.06	-	3.57	-
Magnesium sulfate	7487-88-9	$MgSO_4 \cdot 7H_2O$	246.51	0.36	0.37	0.37
Potassium chloride	7447-40-7	KCl	74.55	0.48	-	-
Calcium chloride	10043-52-4	$CaCl_2 \cdot 2H_2O$	147.02	-	0.10	0.10
**N-source and P-source medium**[1]					**(mmol L^{-1})**	
Ammonium chloride	12125-02-9	NH_4Cl	53.49	3.54	1.43	1.43
Dipotassium hydrogen phosphate	7758-11-4	K_2HPO_4	174.18	0.42	-	-
Potassium dihydrogen phosphate	7778-77-0	KH_2PO_4	136.09	0.21	1.61	0.07
**Additional compounds**[1]					**(mg L^{-1})**	
Allyl-N-thiourea (ATU)[5]	109-57-9	$C_4H_8N_2S$	116.18	-	2	2
Yeast extract[6]	8013-01-2	n.a.	n.a.	-	0.8	-
Peptone[6]	91079-38-8	n.a.	n.a.	-	0.8	-
Casamino acids[6]	65072-00-6	n.a.	n.a.	-	0.8	-
**Trace element solution**[1,7]					**(mL L^{-1})**	
Trace element solution A	-	-	-	0.50	-	-
Trace element solution B	-	-	-	-	0.3	0.3

[1] The synthetic wastewater was prepared by mixing the content of one first 20-L flask containing the C-source medium, and the content of one second 20-L flask containing the N-source and P-source medium, the additional compounds and the trace elements. Each flask was prepared by dissolving salts in demineralized water.
[2] During the feeding phases, the synthetic wastewater of the BC-SBR (BC-SBR) was prepared by diluting the 10-times concentrated media with tap water.
[3] During the feeding phases, the synthetic wastewaters of the PAO-SBR and GAO-SBR were prepared by mixing the 2-times concentrated media in a ratio 1:1.
[4] The BC-SBR was fed with a volumetric OLR of 250 mg_{CODs} cycle^{-1} L_R^{-1}. The PAO-SBR and the GAO-SBR were fed at steady-state with a volumetric OLR of 200 mg_{CODs} cycle^{-1} L_R^{-1}. The PAO-SBR was started with stepwise increase of the volumetric OLR from 15 to 200 mg_{CODs} cycle^{-1} L_R^{-1} in 12 days. The chemical oxygen demand (COD) conversion factor of acetate is 64 g_{COD} mol_{Ac}^{-1}, and of propionate 112 g_{COD} mol_{Pr}^{-1}.
[5] Allyl-N thiourea ($C_4H_8N_2S$) was added in the PAO-SBR and in the GAO-SBR to inhibit nitrification, according to Lopez-Vazquez et al. (2009).
[6] Protein complements were added in the PAO-SBR to sustain the enrichment of *Accumulibacter*, according to the media composition used by diverse authors (Smolders et al., 1994;Hesselmann et al., 1999;Hollender et al., 2002;Schuler and Jenkins, 2003;Zeng et al., 2003;Lu et al., 2006;Lopez-Vazquez et al., 2009;Marcelino et al., 2009). COD conversion factors: 1.4 g_{COD} $g_{yeast\ extract}^{-1}$, 1.4 g_{COD} $g_{casamino\ acids}^{-1}$, 1.2 g_{COD} $g_{peptone}^{-1}$.
7 Two different trace element solutions were used for the BC-SBR (de Kreuk et al., 2005), and for the PAO-SBR and the GAO-SBR (Lopez-Vazquez et al., 2009).

Table SM1.2 Composition of trace element solutions.

Compound	CAS no.	Molecular formula	Molecular weight (g mol^{-1})	Amount per 5 L of stock solution[1] (g)	Concentration in stock solution (mmol L^{-1})
**Trace element solution A (BC-SBR)[1,2]**					
Na_2EDTA[3]	139-33-3	$C_{10}H_{14}N_2O_8Na_2 \cdot 2H_2O$	372.25	637.0	342.2
Zinc sulfate	7733-02-0	$ZnSO_4 \cdot 7H_2O$	287.59	22.0	15.3
Calcium chloride	10043-52-4	$CaCl_2 \cdot 2H_2O$	147.02	81.8	111.3
Manganese chloride	7773-01-5	$MnCl_2 \cdot 4H_2O$	197.92	50.6	51.1
Iron(II) sulfate	7720-78-7	$FeSO_4 \cdot 7H_2O$	278.05	49.9	35.9
Ammonium heptamolybdate	12027-67-7	$(NH_4)_6Mo_7O_{24} \cdot 4H_2O$	1’235.88	16.4	2.7
Copper(II) sulfate	7758-98-7	$CuSO_4 \cdot 5H_2O$	249.71	15.7	12.6
Cobalt(II) chloride	7646-79-9	$CoCl_2 \cdot 6H_2O$	237.96	16.1	13.5
**Trace element solution B (PAO-SBR, GAO-SBR)[1,2]**					
Na_2EDTA[3]	139-33-3	$C_{10}H_{14}N_2O_8Na_2 \cdot 2H_2O$	372.25	50.00	26.86
Zinc sulfate	7733-02-0	$ZnSO_4 \cdot 7H_2O$	287.59	0.60	0.42
Manganese chloride	7773-01-5	$MnCl_2 \cdot 4H_2O$	197.92	0.60	0.61
Iron(III) chloride	7705-08-0	$FeCl_3 \cdot 6H_2O$	270.30	7.50	5.55
Sodium molybdate	7631-95-0	$Na_2MnO_4 \cdot 2H_2O$	205.92	0.30	0.29
Copper(II) sulfate	7758-98-7	$CuSO_4 \cdot 5H_2O$	249.71	0.15	0.12
Cobalt(II) chloride	7646-79-9	$CoCl_2 \cdot 6H_2O$	237.96	0.75	0.63
Boric acid	11113-50-1	H_3BO_3	61.83	0.75	2.43
Potassium iodide	7681-11-0	KI	166.00	0.90	1.08

[1] The compositions of the trace element solutions A and B were taken from de Kreuk et al. (2005) and Lopez-Vazquez et al. (2009), respectively.
[2] Each trace element solution was prepared in demineralized water.
[3] Disodium ethylenediaminetetraacetate.

Supplementary material 2

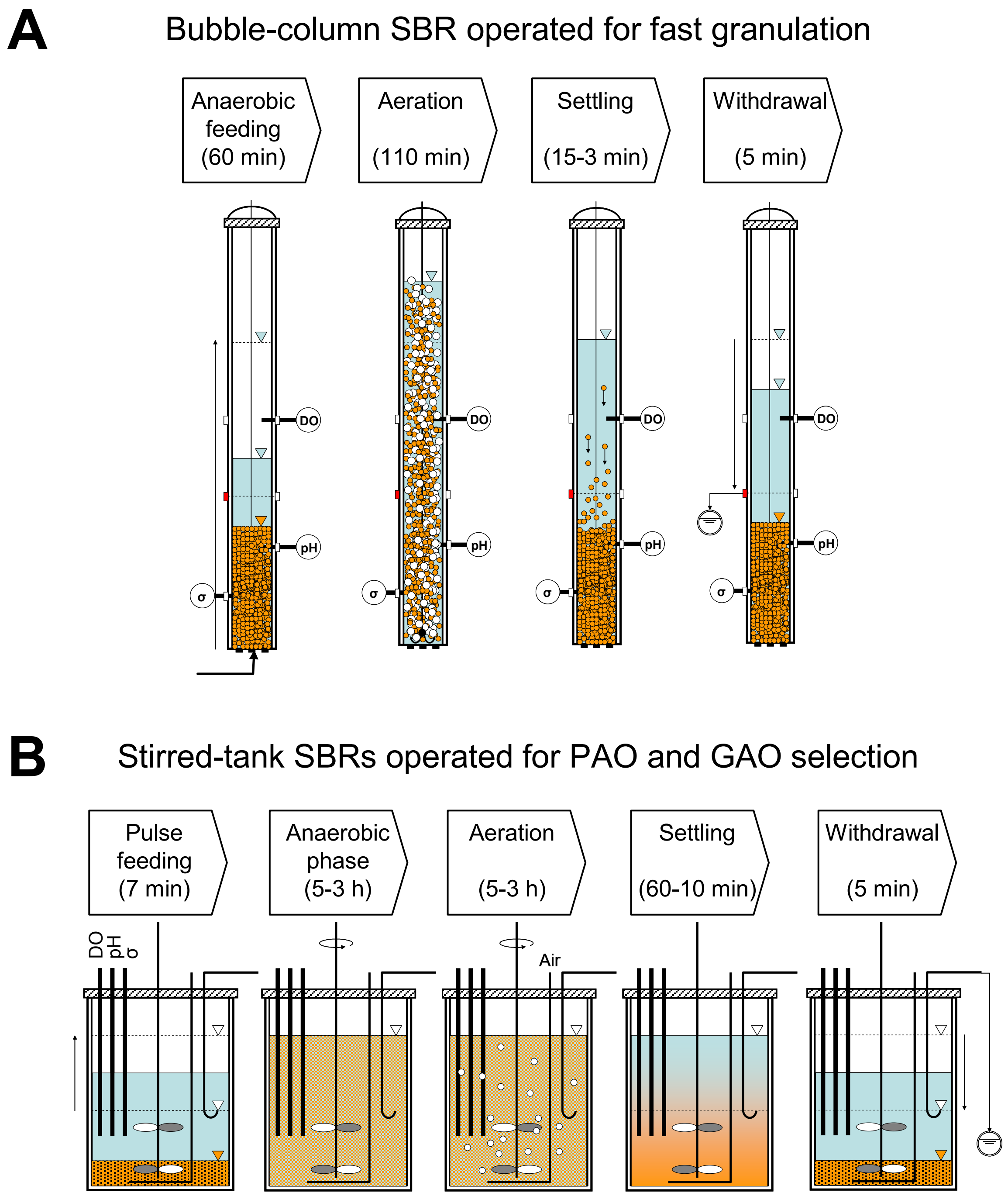

Figure SM2.1 Schemes of the bubble-column and stirred-tank SBRs. The bubble-column SBR design **(A)** was used in combination with wash-out conditions for fast cultivation of granules. The stirred-tank SBR design **(B)** was used in combination with steady-state conditions and full control of anaerobic and aerobic phases for the cultivation of enrichments of PAO and GAO.

Supplementary material 3

Table SM3.1 Efficiency of fluorescent dyes for investigating flocs and granules structures by CLSM.

Fluorescent dyes and specifications		CLSM signal recording		Cell and biofilm specificity[3]	Staining and imaging result
(excitation/emission in nm, fluorescence color)		Excitation (nm)[1]	Emmission (nm)[2]		
Reflection signal	(no staining)	483	475-495	Overall aggregate structure	Strong signal
Autofluorescence	(no staining)	483	475-495	Overall aggregate structure	Weak signal
Propidium iodide	(PI, 535/617, red)	483 and 536	475-495 and 580-650	Nucleic acids	Staining OK
SYBR Green	(497/520, green)	494	479-505 and 510-550	Nucleic acids	Staining OK, incubation over night
SYPRO Red	(550/630, red)	485 and 550	475-495 and 560-700	Nucleic acids	Staining OK, incubation over night
SYTO 9	(485/500, green)	483 and 536	475-495 and 580-650	Nucleic acids	Weak signal
SYTO 60	(652/678, red)	490 and 652	485-495 and 665-750	Nucleic acids	Staining OK
SYTOX Green	(504/523, green)	504	597-613 and 510-560	Nucleic acids	Staining OK
Rhodamine 6G	(528/551, green)	485 and 525	475-495 and 535-600	Cells, overall aggregate structure	Strong cell and matrix staining
FM 1-43	(472/580, green)	480	470-490 and 550-650	Membrane lipids	Staining OK
FM 4-64	(515/640, red)	480 and 545	470-490 and 590-700	Membrane lipids	Staining OK
Nile Red	(552/636, red)	480 and 545	470-490 and 590-700	Hydrophobic regions	Staining OK
DDAO	(646/659, red)	485 and 625	475-495 and 640-750	Extracellular DNA	Staining OK

[1] Laser excitation wavelength used to target reflection signal (first wavelength) and to excite the fluorescent dye (second wavelength). When only one value is given, this wavelength was used to target both reflection and fluorochrome excitation.
[2] Wavelength ranges used to collect the emitted reflection signal (first range) and fluorescence signal (second range).
[3] Specificity of the fluorescent dye for internal and external cell components, as well as biofilm components, according to Life Technologies (2012).

Table SM3.2 Efficiency of lectins for targeting glycoconjugates in the matrices of granular biofilms by means of CLSM.

Letter code for lectins used	Origin	Fluorescent label[1]	CLSM signal recording		Sugar specificity[3]	Biomass staining result
			Excitation (nm)[2]	Emission (nm)[2] and color allocation		
LTL	*Lotus tetragonolobus*	FITC	488	480-500, gray	α-Fuc	Only partial staining
AAL	*Aleuria aurantia*	Alexa 488		500-560, green	Fuc, Fuc-α-6-GlcNAc	Direct binding to cell surface
IAA	*Iberis amara*	Alexa 488			α-Gal, GlcNAc	Embedded continuous matrix, specific binding[7]
HAA	*Helix aspersa*	FITC			α-GalNAc	Matrix surrounding larger clusters, filamentous bacteria
SBA	*Glycine max*	FITC			α-GalNAc, β-GalNAc	Matrix surrounding larger clusters
PNA	*Arachis hypogaea*	FITC			α-Gal, β-GalNAc, Gal-β-3-GalNAc	Direct binding to cell surface
ECL	*Erythrina cristagalli*	FITC			α-Gal, β-Gal, β-GlcNAc, Gal-β-4GlcNAc	Direct binding to cell surface, not adequate staining
ConA	*Canavalia ensiformis*	FITC			α-Glc, α-Man	Direct binding to cell surface
LcH	*Lens culinaris*	FITC			α-Glc, α-GlcNAc, α-Man	Matrices of microcolony interface
WGA	*Triticum vulgaris*	FITC			β-GlcNAc, sialic acid	Matrices of microcolony interface
LEA	*Lycopersicon esculentum*	FITC			β-GlcNAc, $(GlcNAc)_{2-4}$	Matrix surrounding larger clusters, filamentous bacteria
STA	*Solanum tuberosum*	FITC			$(GlcNAc)_{2-4}$	Embedded continuous matrix
PHA-L	*Phaseolus vulgaris*	FITC			Complex sugar[6]	Embedded continuous matrix

[1] All lectins were labeled with green fluorescent dyes, namely Alexa Fluor 488 or fluorescein isothiocyanate (FITC). Alexa Fluor 488 is more photostable than FITC.
[2] One wavelength was used to target for the reflection signal as well as for the fluorochrome excitation. Two wavelength ranges were used to collect the reflection signal (first range) and the emitted fluorescence (second range). The same set points were used for each labeled lectin.
[3] Specificity of the lectin for sugar residues according to Oawa and Tsuji (1987), Zippel and Neu (2011), and manufacturer's specifications of EY Laboratories (USA) and Vectorlabs (2012). Legend: Fuc, L-fucose; Gal, D-galactose; GalNAc, N-acetylgalactosamine; Glc, D-glucose; GlcNAc, N-acetylglucosamine; Man, mannose.
[6] Complex sugar with structure Galβ4GlcNAcβ6(GlcNAcβ2Manα3)Manα3
[7] Lectin binding to specific types of cell clusters.

Supplementary material 4

Table SM4.1 Targeted 16S rRNA oligonucleotide probes used to follow by FISH-CLSM the temporal and spatial evolutions of *Zoogloea*, *Accumulibacter*, *Competibacter*, and ammonium-oxidizing organisms (AOO) during granule formation.

Probe name[1,2]	Full name[1]	Accession number[1]	Target organisms[1,2]	Sequence 5'-3' [1,2]	FA[1,2] (%)	Reference[1,2]
ZOGLO-647 (or ZRA23a)	S-*-Zoglo-0647-a-A-18	pB-00332	Most *Zoogloea* affiliates	CTG CCG TAC TCT AGT TAT	35	(Rossello-Mora *et al.*, 1995)
ZOGLO-1416	S-G-Zoglo-1416-a-A-18	pB-00873	*Zoogloea* spp.	TCT GGT AAA CCC CAC TCC	**25**	(Loy *et al.*, 2005)
PAO-462	n.a.	pB-00910	Most *Accumulibacter*	CCG TCA TCT ACW CAG GGT ATT AAC	35	(Crocetti *et al.*, 2000)
PAO-651 (or ACCBA651)	S-G-Accba-0651-a-A-18	pB-00774	Most *Accumulibacter*	CCC TCT GCC AAA CTC CAG	35	(Crocetti *et al.*, 2000)
PAO-846	S-S-Rhodo-0846-a-A-18	pB-00623	Most *Accumulibacter*	GTT AGC TAC GGC ACT AAA AGG	35	(Crocetti *et al.*, 2000)
GAO-Q431	n.a.	pB-00775	Some *Competibacter*	TCC CCG CCT AAA GGG CTT	35	(Crocetti *et al.*, 2002)
GAO-Q989 (or GB-G1)	n.a.	pB-00967	Some *Competibacter*	TTC CCC GGA TGT CAA GGC	35	(Crocetti et al., 2002;Kong et al., 2002)
GB-G2	n.a.	pB-00968	Some *Competibacter*	TTC CCC AGA TGT CAA GGC	35	(Kong *et al.*, 2002)
NSO-190 [3]	S-F-bAOB-0189-a-A-19	pB-00249	Betaproteobacterial AOO	CGA TCC CCT GCT TTT CTC C	**55**	(Mobarry *et al.*, 1996)

[1] Information on oligonucleotide probes provided by probeBase (Loy *et al.*, 2003).
[2] Information on oligonucleotide probes provided in the FISH Handbook for Biological Wastewater Treatment (Nielsen *et al.*, 2009).
[3] The probe NSO190 has been reported to diplay similar coverage as the probe NSO1225, but with more reliability in probe brightness (Nielsen *et al.*, 2009).

The ZOGLO and PAO probes were labeled with the cyanine fluorescent dye Cy3. The GAO probes were labeled with the fluorescent dye ATTO488. The NSO probe was labeled with the fluorescein 6-FAM. The following wavelength ranges were used to record CLSM signals from hybridized bioaggregate samples. Cy3: excitation 550 nm, reflection signal 540-560 nm, and Cy3 emission 565-630 nm. ATTO488: excitation 501 nm, reflection signal 496-506 nm, and ATTO488 emission 515-580 nm. 6-FAM: excitation 495 nm, reflection signal 490-500, and 6-FAM emission 505-565 nm. Even though the ZOGLO-1416 and ZOGLO-647 probes require different stringencies, hybridization was successfully tested for each individual probes with a stringency of 35%, and thus both probes were also used in a mixture. The photostable ATTO488 dye did only exhibited weak fluorescence, but digital images were still interpretable. CLSM analyses with the photosensitive 6-FAM dye were performed with reduced exposition times. All oligonucleotide probes were ordered by Invitrogen (Life Technologies Europe B.V., Switzerland).

Supplementary material 5

Table SM5.1 Closest phylogenetic bacterial affiliations of OTUs detected in the **BC-SBR**, that were obtained with the pyrosequencing-based bioinformatics PyroTRF-ID procedure (Weissbrodt and Shani, et al., paper accepted).

T-RF[1] (bp)	Sample[2]	Counts[3] (-)	Fraction of T-RF[4] (%)	Affiliation[5] Phylum → Order	Family → Genus	Accession number[5]	Closest relative and original microbiota[6]	Smith-Waterman mapping score (-)[7] Absolute	Normalized
32	BC-II	276	35	O: *Xanthomonadales*	G: *Thermomonas*	EU834762<br>AB355702	Uncultured bacterium clone K81 from lab-scale EBPR activated sludge<br>*Thermomonas brevis* S47 sequence from consortia exhibiting intergeneric coaggregation	425	0.983
		128	16	O: *Xanthomonadales*	G: *Pseudoxanthomonas*	AY512829	*Pseudoxanthomonas mexicana* from the rhizosphere of Alyssum murale	385	0.955
		111	14	C: *Gammaproteobacteria*	O: *Xanthomonadaceae*	AY212636	Uncultured bacterium clone 184up from streams contaminated with fecal material	386	0.960
		32	4	O: *Xanthomonadales*	G: *Dokdonella*	AM981200	*Dokdonella* sp. RaM5-2	349	0.881
		3	0.4	O: *Xanthomonadales*	G: *Stenotrophonomas*	AY259519	*Stenotrophomonas* sp. FB206 isolated from cooked vegetables	322	0.839
		88	11	O: *Flavobacteriales*	G: *Sejongia*	AY468464	*Chryseobacterium* sp. FRGDSA 4034/97 isolated from skin ulcer and rainbow trout	434	1.000
		11	1.3	O: *Flavobacteriales*	F: *Cryomorphaceae*	EU803886	Uncultured bacterium clone 5C231539 from Lake Gatun	320	0.779
		7	0.9	O: *Flavobacteriales*	G: *Kaistella*	AF502204	Uncultured bacterium clone HP1B06 from EBPR sludge	339	0.921
		29	3.7	O: *Rhodobacterales*	G: *Thioclava*	CU919741	Uncultured *Alphaproteobacterium* clone QEDV3CF12 from anaerobic digester	401	0.973
		17	1.2	O: *Rhodobacterales*	G: *Rhodobacter*	AY212706	Uncultured bacterium clone 257ds10 from streams contaminated with fecal material	448	1.000
		37	5	O: *Rhodocyclales*	G: *Rhodocyclus*	AF502230	Uncultured bacterium clone HP1A31 from lab-scale EBPR sludge	363	0.917
62	BC002	22	67	O: *Actinomycetales*	G: *Tessaracoccus*	GQ097568	Uncultured bacterium clone nbw397a07c1 from human skin microbiome	380	0.977
		10	30	P: candidate phylum TM7		EU104134	Uncultured bacterium clone M0509_49 from activated sludge	373	0.912
72	BC002	18	40	O: *Rhodocyclales*	G: *Zoogloea*	AJ011506	*Zoogloea resiniphilia* strain DhA-35T	376	0.962
		13	35	O: *Rhodocyclales*	G: *Thauera*	AY945909	Uncultured bacterium clone DR-48 from denitrifying bioreactor	348	0.926
	BC059	11	100	O: *Rhodocyclales*	G: *Zoogloea*	AJ011506	*Zoogloea resiniphilia* strain DhA-35T	373	0.942
180	BC002	4	100	P: *Acidobacteria*		GQ396818	Uncultured bacterium clone AK1AB1_04A from recently-deglaciated soils	323	0.807
185	BC002	8	89	O: *Rhizobiales*		FJ719048	Uncultured bacterium clone p03_D09 from aquifer sediments	296	0.757
		1	11	O: *Rhodobacterales*		FJ719099	Uncultured bacterium clone p04_H05 from aquifer sediments	318	0.941
	BC059	14	100	O: *Rhizobiales*		AF502218	Uncultured bacterium clone HP1B02 from EBPR sludge	409	0.969
190	BC002	20	49	O: *Rhizobiales*		CU918969	Uncultured *Alphaproteobacterium* clone QEEA1DD02 from anaerobic digester	404	0.962
		18	44	O: *Burkholderiales*	G: *Acidovorax*	EU539550	Uncultured bacterium clone nbt241e12 from human skin microbiota	356	0.944
193	BC002	22	30	O: *Burkholderiales*	G: *Acidovorax*	EU375646	*Acidovorax* sp. u41 from bacterial community degrading organic pollutants	363	0.936
		21	28	O: *Burkholderiales*	G: *Simplicispira*	AJ505861	*Comamonadaceae* bacterium strain PIV-16-2 from denitrifying bacterial community	377	0.969
		13	17	O: *Burkholderiales*	G: *Delftia*	EF515241	Uncultured bacterium clone 21g10 from electricigen enrichment in a MFC	355	0.920
	BC059	72	94	O: *Burkholderiales*	G: *Acidovorax*	AJ864847	*Acidovorax* sp. strain J33 from high mountain lake habitats	383	0.923
		5	6	O: *Xanthomonadales*		EU662583	Uncultured bacterium clone MC1_16S_13 from sulfidic karst system	343	0.868
	BC-II	10	91	O: *Burkholderiales*	G: *Acidovorax*	AJ864847	*Acidovorax* sp. J33 isolated from biofilm on granit stone	384	1.000
195	BC002	231	84	O: *Rhodocyclales*	G: *Zoogloea*	AF234684	Uncultured sludge bacterium H7 from nitrifying-denitrifying industrial WWTP	385	0.990
		18	7	O: *Pseudomonadales*	G: *Acinetobacter*	GQ073520	Uncultured bacterium clone nbw209f04c1 from human skin microbiome	337	0.988
	BC059	4793	100	O: *Rhodocyclales*	G: *Zoogloea*	EU639144	Uncultured bacterium clone LPB19 from EBPR sludge	402	0.854
	BC-II	6	40	O: *Burkholderiales*	G: *Aquamonas*	DQ337068	Uncultured bacterium clone EV818SWSAP25 from subsurface water of the Kalahari Shield	361	0.919
		5	33	O: *Xanthomonadales*	G: *Pseudoxanthomonas*	GU122958	*Pseudoxanthomonas* sp. DBTS3 isolated from aquaculture	288	0.742
		3	20	C: *Alphaproteobacteria*	F: *Rhodospirillaceae*	AF527585	Uncultured bacterium clone LPB46 from lab-scale EBPR sludge	333	0.902
		1	7	O: *Rhodocyclales*	G: *Zoogloea*	EU834814	Uncultured bacterium clone OTUc87 from an aerobic phosphorus removal process	303	0.953
198	BC002	65	94	P: candidate phylum TM7		DQ640696	Uncultured TM7 bacterium clone Skagenf80 from EBPR-WWTP	367	0.870
200	BC-II	3	100	O: *Rhodocyclales*	G: *Rhodocyclus*	AF204247	Uncultured *Betaproteobacterium* SBRB34 sequence from full-scale EBPR sludge	314	0.858

Table SM5.1 – *Continued*

T-RF[1] (bp)	Sample[2]	Counts[3] (-)	Fraction of T-RF[4] (%)	Affiliation[5] Phylum → Order	Family → Genus	Accession number[5]	Closest relative and original microbiota[6]	Smith-Waterman mapping score (-)[7] Absolute	Normalized
201	BC002	10	48	P: *Chloroflexi*		CU927307	Uncultured *Chloroflexi* bacterium clone EDN7BB07 from anaerobic digester	443	1.000
		8	38	O: *Xanthomonadales*	F: *Xanthomonadaceae*	FJ612198	Uncultured bacterium clone DP3.5.36 from lake ecosystem	360	0.923
206	BC-II	6	100	O: *Sphingobacteriales*		EF019693	Uncultured *Bacteroidetes* bacterium clone Elev_16S_854 from aspen rhizosphere	387	0.896
208	BC002	6	30	O: *Burkholderiales*	G: *Rhodoferax*	AB154311	Uncultured bacterium clone S9F-53 from eutrophic lake	351	0.931
		1	10	O: *Burkholderiales*	G: *Sphaerotilus*	AB087568	*Sphaerotilus* sp. L19 from filamentous activated sludge bulking process	374	0.979
210	BC002	6	42	O: *Acidobacteriales*		FJ230900	Uncultured bacterium clone F25 from river water receiving antibiotics-rich effluents	403	0.988
		5	35	P: *Firmicutes*	G: *Trichococcus*	EU234209	Uncultured bacterium clone B14 from river water receiving antibiotics-rich effluents	295	1.000
211	BC059	7	54	O: *Burkholderiales*	F: *Comamonadaceae*	EF540425	Uncultured soil bacterium clone MK4a from semi-coke	345	0.925
		6	46	O: *Rhodocyclales*	F: *Rhodocyclaceae*	DQ088735	Uncultured bacterium clone BE16FW031601GDW_hole1-9 from gold mine groundwater	343	0.724
213	BC002	17	73	O: *Burkholderiales*	F: *Comamonadaceae*	AY662010	Uncultured bacterium clone 300A-D08 from groundwater contaminated with nitric acid	365	0.979
	BC-II	11	92	O: *Rhodocyclales*	G: *Rhodocyclus*	AF502230	Uncultured bacterium clone HP1A31 from lab-scale EBPR sludge	356	0.942
214	BC002	136	48	O: *Rhodocyclales*	G: *Dechloromonas*	AY064177	Uncultured *Betaproteobacterium* clone UCT N123 from EBPR-WWTP	382	0.977
		45	16	O: *Burkholderiales*	G: *Rhodoferax*	AB452981	*Betaproteobacterium* clone HIBAF001 from freshwater bacterioplankton	366	0.948
		29	10	O: *Rhodocyclales*	G: *Methyloversatilis*	AY436796	*Methyloversatilis universalis* strain EHg5 isolated from sediments	364	0.958
		12	4	O: *Rhodocyclales*	G: *Zoogloea*	DQ413172	*Zoogloea* sp. EMB 357 isolated from anaerobic-aerobic SBR	344	0.901
		11	4	O: *Burkholderiales*	G: *Aquamonas*	DQ521469	Uncultured bacterium clone ANTLV1_A07 from Antarctica lake ice cover microbiota	366	0.951
		4	1	O: *Rhodocyclales*	G: *Rhodocyclus*	EF565151	Uncultured bacterium clone VIR_D5 from EBPR sludge rich in *Accumulibacter*	368	0.981
	BC059	14	88	O: *Rhodocyclales*	G: *Dechloromonas*	DQ413103	Uncultured bacterium clone 44 from anaerobic-aerobic SBR	381	0.890
		2	12	O: *Rhodocyclales*	G: *Rhodocyclus*	AF502224	Uncultured bacterium clone HP1A13 from EBPR sludge	372	0.923
	BC-II	769	99.6	O: *Rhodocyclales*	G: *Rhodocyclus*	AB200295<br>AF502230	Uncultured bacterium clone UTFS-OF09-d22-93 from lab-scale EBPR system<br>Uncultured bacterium clone HP1A31 from lab-scale EBPR sludge	371	0.949
		1	0.1	O: *Rhodocyclales*	G: *Dechloromonas*	DQ413103	Uncultured bacterium clone 44 form EBPR sludge	321	0.988
		1	0.1	O: *Rhodocyclales*	G: *Methyloversatilis*	DQ066958	Uncultured bacterium clone pLW-7 from sediments of Lake Washington	368	0.958
		1	0.1	O: *Nitrosomonadales*	G: *Nitrosomonas*	EU937892	Uncultured bacterium clone 3BR-6DD from riparian iron oxidizing biofilm	278	0.753
215	BC002	14	44	O: *Rhodocyclales*	G: *Methyloversatilis*	DQ066958	Uncultured bacterium clone pLW-7 from sediment consortium metabolizing C1 compounds	349	0.928
		9	28	P: *Chloroflexi*	G: *Caldilinea*	CU917747	Uncultured *Chloroflexi* bacterium clone QEEB2DA06 from anaerobic digester	279	0.730
		2	6	O: *Rhodocyclales*	G: *Rhodocyclus*	FJ719063	Uncultured bacterium clone p04_A04 from aquifer sediments	320	0.938
		1	3	O: *Rhodocyclales*	G: *Dechloromonas*	AY062126	Uncultured *Betaproteobacterium* clone UCT N141 from EBPR-WWTP	306	0.820
	BC-II	5	31	O: *Rhodocyclales*	G: *Rhodocyclus*	AF502230	Uncultured bacterium clone HP1A31 from lab-scale EBPR sludge	337	0.864
		2	13	O: *Rhodocyclales*	G: *Methyloversatilis*	GQ340363	Uncultured bacterium clone VE08-161-BAC from a drinking water resevoir	298	0.914
		1	6	O: *Nitrosomonadales*	G: *Nitrosomonas*	GQ396862	Uncultured bacterium clone AK1AB2_05E from deglaciated soil	278	0.788
216	BC002	5	35	O: *Rhodocyclales*	F: *Rhodocyclaceae*	NR029035	*Quatrionicoccus australiensis* strain Ben 117 from activated sludge	311	0.881
		3	21	O: *Burkholderiales*	F: *Comamonadaceae*	EU180529	*Betaproteobacterium* BAC49 from granular activated carbon filters	273	0.853
		1	7	O: *Nitrosomonadales*	G: *Nitrosomonas*	EU937892	Uncultured bacterium clone 3BR-6DD from an iron oxidizing freshwater habitat	348	0.909
	BC-II	8	62	O: *Rhodocyclales*	G: *Methyloversatilis*	CU922545	Uncultured *Betaproteobacterium* clone QEDR3BH07 from anaerobic digester	361	0.914
		2	15	C: *Anaerolineae*		EU104216	Uncultured bacterium clone N1512_47 from activated sludge	269	0.727
		1	8	O: *Rhodocyclales*	G: *Rhodocyclus*	AF502230	Uncultured bacterium clone HP1A31 from lab-scale EBPR sludge	276	0.865
		1	8	O: *Rhodocyclales*	F: *Rhodocyclaceae*	GU454920	Uncultured bacterium clone ambient_uncontrolled-59 16S from process of alkaline fermentation of waste activated sludge producing short-chain fatty acids	347	0.938
217	BC002	15	46	O: *Actinomycetales*		AF513101	Uncultured bacterium clone 7 from foaming activated sludge	385	0.955
		12	36	O: *Rhodocyclales*	G: *Thauera*	AM084110	*Thauera* sp. R-28312 from denitrifying sludge	386	1.000
	BC-II	1	33	O: *Nitrosomonadales*	G: *Nitrosomonas*	EU937892	Uncultured bacterium clone 3BR-6DD from riparian iron oxidizing biofilm	295	0.764
		1	33	O: *Rhizobiales*	G: *Devosia*	AF236010	*Betaproteobacterium* A0640	298	0.859
		1	33	O: *Burkholderiales*	G: *Ideonella*	GQ472390	Uncultured bacterium clone 4D3-5 from sediments of shallow Lake Dongping	207	0.758

Table SM5.1 – *Continued*

T-RF[1] (bp)	Sample[2]	Counts[3] (-)	Fraction of T-RF[4] (%)	Affiliation[5] Phylum → Order	Family → Genus	Accession number[5]	Closest relative and original microbiota[6]	Smith-Waterman mapping score (-)[7] Absolute	Normalized
220	BC-II	48	89	O: *Rhizobiales*	G: *Aminobacter*	NR025302	*Aminobacter niigataensis* strain DSM7050	448	1.000
223	BC002	545	99	O: *Actinomycetales*	G: *Tetrasphaera*	AF255629	Uncultured bacterium clone Ebpr19 from EBPR-WWTP	374	0.944
	BC059	23	100	O: *Actinomycetales*	G: *Tetrasphaera*	AF527583	Uncultured bacterium clone LPB21 from EBPR sludge	371	0.949
	BC-II	44	72	O: *Actinomycetales*	F: *Intrasporangiaceae*	AF255629	Uncultured bacterium clone Ebpr19 from EBPR sludge	373	0.961
		15	25	O: *Rhodobacterales*	F: *Hyphomonadaceae*	AF236001	*Alphaproteobacterium* A0904	298	0.674
224	BC059	135	96	O: *Rhodobacterales*	F: *Hyphomonadaceae*	AF236001	*Alphaproteobacterium* A0904	285	0.625
228	BC002	50	88	O: *Actinomycetales*	F: *Intrasporangiaceae*	AF513091	Uncultured bacterium clone 17 from faming activated sludge	382	0.946
		3	5	C: *Actinobacteria*	F: *Microthrixaceae*	CU917839	Uncultured *Actinobacterium* clone QEEB1AC11 from anaerobic digester	388	0.965
232	BC-II	5	71	C: *Alphaproteobacteria*	F: *Rhodospirillaceae*	DQ066972	Uncultured bacterium clone pLW-38 from sediments of Lake Washington	259	0.738
233	BC002	26	87	P: candidate phylum TM7		FJ534960	Uncultured bacterium clone 14 from anaerobic fermentation of waste activated sludge	271	0.666
		2	7	O: *Phycisphaerales*		FJ612210	Uncultured bacterium clone DP7.3.10 from lake ecosystem	283	0.625
237	BC-II	17	94	C: *Gammaproteobacteria*		AF361092	Uncultured bacterium clone SBRQ157 from GAO enrichment	324	0.883
238	BC-II	272	99	C: *Gammaproteobacteria*		FJ356056<br>AF361096	Uncultured bacterium clone G112 from lab-scale EBPR system<br>Uncultured bacterium clone SBRH10 from GAO enrichment	446	0.982
247	BC-II	3	100	O: *Rhodocyclales*	G: *Rhodocyclus*	AB200295	Uncultured bacterium clone UTFS-OF09-d22-93 from lab-scale EBPR sludge	305	0.762
250	BC002	35	92	O: *Pseudomonadales*	G: *Acinetobacter*	EU467673	Uncultured bacterium clone molerat_2g12_1 from gut microbiota	415	0.883
252	BC002	4	80	O: *Sphingobacteriales*		FJ793188	Uncultured bacterium clone TDB87 from a hot spring dam	295	0.905
	BC-II	4	80	O: *Sphingobacteriales*		DQ984594	Uncultured bacterium clone IYF104 from Mt. Nan-Jen litterfall	349	0.928
253	BC002	14	88	O: *Ignavibacteriales*	F: *Ignavibacteriaceae*	AB186808	Uncultured bacterium from polychlorinated-dioxin-dechlorinating microbial community	462	0.977
	BC-II	7	100	O: *Sphingobacteriales*		AM411964	*Sphingobacterium* sp. P-7 isolated from soil	355	0.989
257	BC002	7	58	O: *Sphingobacteriales*		EF562554	Uncultured bacterium clone CC_3 from consortium degrading complex organic matter	380	0.997
		5	42	P: candidate phylum TM7		AB200304	Uncultured bacterium clone UTFS-OF09-d22-29 from EBPR-WWTP	283	0.663
	BC-II	7	100	O: *Sphingobacteriales*		EU283377	Uncultured *Bacteroidetes* bacterium clone AS56 from aerated MBR treating wastewater	267	0.914
258	BC-II	16	93	O: *Nitrospirales*	G: *Nitrospira*	AF314422	Uncultured bacterium PHOS-HE34 from an aerobic EBPR ecosystem	389	0.982
259	BC-II	38	97	O: *Sphingobacteriales*		EU104185	Uncultured bacterium clone N1512_12 from activated sludge	267	0.706
		1		O: *Nitrospirales*	G: *Nitrospira*	GQ487996	Uncultured bacterium clone V8-58 from heavy metal polluted soil	319	0.788
260	BC002	16	76	O: *Nitrospirales*	G: *Nitrospira*	AF314422	Uncultured bacterium PHOS-HE34 from an aerobic EBPR ecosystem	366	0.924
		4	19	O: *Sphingobacteriales*		FJ660602	Uncultured bacterium clone A194 from full-scale WWTP	334	0.859
	BC059	3	100	O: *Sphingobacteriales*		AY302128	Uncultured bacterium clone DSBR-B082 from denitrifying community	354	0.878
264	BC059	3	100	O: *Thiotrichales*	G: *Thiothrix*	L79963	*Thiothrix fructosivorans* strain I, a filamentous sulfur bacterium from WWTP	334	0.933
277	BC-II	3	100	P: *Spirochaetes*	F: *Leptospiraceae*	AY293856	*Turneriella parva serovar* Parva strain H	364	0.963
281	BC-II	4	100	C: *Flavobacteria*		CU925607	Uncultured *Bacteroidetes* bacterium clone QEDN8AF04 from anaerobic digester	321	0.870
289	BC002	4	57	O: *Sphingomonadales*	G: *Sphingobium*	AB040739	Sphingobium cloacae a nonylphenol-degrading bacterium isolated from WWTP	277	0.785
		3	43	O: *Rhodospirillales*	F: *Rhodospirillaceae*	EU864465	Uncultured bacterium clone E52 from river water receiving antibiotics-rich effluents	350	0.967
290	BC-II	1	100	O: *Rhodospirillales*	F: *Rhodospirillaceae*	AM935307	Uncultured *Alphaproteobacterium* clone AMEC9 from hydrocarbon-contaminated soil	358	1.000
294	BC002	1	100	O: *Clostridiales*	G: *Ruminococcus*	DQ796981	Uncultured bacterium clone RL386_aao85c11 from human gut microbiome	289	0.906
298	BC-II	7	88	C: *Gammaprotebacteria*		AB255053	Uncultured bacterium gene clone IC-7 from corroded concrete sample	209	0.565

Table SM5.1 – *Continued*

T-RF[1] (bp)	Sample[2]	Counts[3] (-)	Fraction of T-RF[4] (%)	Affiliation[5]		Accession number[5]	Closest relative and original microbiota[6]	Smith-Waterman mapping score (-)[7]	
				Phylum → Order	Family → Genus			Absolute	Normalized
302	BC002	6	75	C: *Anaerolineae*		EU332818	Uncultured organism clone OTUI177 from aerobic EBPR-SBR	310	0.831
304	BC002	28	93	C: *Gammaproteobacteria*		FJ356049	Uncultured bacterium clone G5 from lab-scale EBPR system from anaerobic digester	383	0.844
306	BC-II	38	97	P: *Armatimonadetes*		CU921283	Uncultured unclassified bacterium clone QEDS2BB03	218	0.472
318	BC-II	17	100	O: *Sphingobacteriales*	G: *Cytophaga*	EU104191	Uncultured bacterium clone N1512_18 from activated sludge	196	0.525
325	BC002	4	100	O: *Sphingobacteriales*		GQ396974	Uncultured bacterium clone AK1DE1_04E from recently-deglaciated soils	299	0.779
392	BC-II	33	100	C: *Deltaproteobacteria*	G: *Bdellovibrio*	CU466777	Uncultured bacterium sequence from anoxic basin of a municipal WWTP	262	0.663
399	BC059	12	100	O: *Rhodocyclales*	G: *Dechloromonas*	EF632559	*Dechloromonas* sp. A34, a bacterium from phosphate mining overburden respiring selenate	378	0.922

[1] Size of target terminal-restriction fragments (T-RF) obtained with *Hae*III digestion and forming operational taxonomic units (OTU).
[2] Original grab biomass samples taken on days 2 (flocculent sludge) and 59 (early-stage granules) in the BC-SBR and selected for pyrosequencing analysis. The pyrosequencing dataset of a third biomass sample originating from a previous bubble-column SBR (BC-II) was used to affiliate the OTUs detected at mature stage. The BC-II sample comprised mature AGS that was maintained on long term (2 years) under steady-state conditions, and that displayed similar T-RFLP profile than the mature AGS obtained at mature stage in the BC-SBR.
[3] Number of sequences from the pyrosequencing dataset that were related to the particular reference organism.
[4] Different bacterial populations can contribute to the same T-RF. The percentage of contribution of each population to the target T-RF is given in this column.
[5] Closest bacterial affiliations and GenBank accession numbers obtained after mapping against the Greengenes reference sequences of 16S rRNA encoding gene (McDonald *et al.*, 2012). Predominant OTUs present in the bacterial communities are highlighted in bold font. Legend: P = phylum, C = class, O = order, F = family, G: genus.
[6] Description of closest relatives and original microbiota from which the reference clones were isolated were obtained after submitting the accession numbers into the GenBank public database (Benson *et al.*, 2011).
[7] The Smith-Waterman (SW) score was used as mapping similarity measure. SW scores consider nucleotide positions and gaps in the sequence structures. The highest absolute SW score that can be obtained is the length of the sequence itself. Each absolute SW score was normalized to the length of the related denoised centroid sequence in order to allow comparison between sequences of various lengths. After mapping in MG-RAST (Meyer *et al.*, 2008), the affiliations obtained for the two denoised pyrosequencing datasets were related to traditional sequence identity scores of 99.7±0.5%.

Table SM5.2 Closest phylogenetic bacterial affiliations of OTUs detected in the **PAO-SBR** obtained with PyroTRF-ID.

T-RF[1] (bp)	Sample[2]	Counts[3] (-)	Fraction of T-RF[4] (%)	Affiliation[5] Phylum → Order	Family → Genus	Accession number[5]	Closest relative and original microbiota[6]	Smith-Waterman mapping score (-)[7] Absolute	Normalized
32	PAO109	208	44	C: *Gammaproteobacteria*	F: *Xanthomonadaceae*	EU834761	Uncultured bacterium clone K71 from EBPR activated sludge	407	0.875
		160	34	P: *Bacteroidetes*	F: *Flavobacteriaceae*	AF502206	Uncultured bacterium clone HP1B29 from EBPR sludge	395	0.956
		32	7	C: *Alphaproteobacteria*	F: *Rhodobacteraceae*	CU924686	Uncultured *Alphaproteobacterium* clone QEDP1DH11 from mesophilic digester	434	0.964
58		6	50	P: *Gemmatimonadetes*	G: *Gemmatimonas*	AP009153	*Gemmatimonas aurantiaca* T-27	368	0.944
		4	33	O: *Phycisphaerales*		AY957928	Uncultured bacterium clone B3NR69D13 from drinking water biofilm	165	0.448
		2	17	P: *Armatimonadetes*		GQ263883	Uncultured bacterium clone WC2_131 from a simulated radioactive waste site	157	0.376
64		2	100	O: *Burkholderiales*	G: *Hydrogenophaga*	AB300163	*Hydrogenophaga* sp. AH-24 gene	362	0.916
176		2	100	P: *Verrucomicrobia*	F: *Opitutaceae*	EF018451	Uncultured proteobacterium clone Amb_16S_1076 from Aspen soil	299	0.757
183		3	100	O: *Xanthomonadales*	G: *Pseudoxanthomonas*	DQ376568	Uncultured bacterium clone 46-ORF03 from an aerobic EBPR process	229	0.571
185		9	100	O: *Rhodobacterales*	F: *Hyphomonadaceae*	AJ617876	Uncultured bacterium 16S rRNA gene from paddy soil	371	0.961
186		7	100	O: *Rhizobiales*		FM209362	Uncultured bacterium partial 16S rRNA gene from Negev desert sand	300	0.723
188		10	40	O: *Rhizobiales*	F: *Bradyrhizobiaceae*	AJ300771	*Afipia* sp. LMG 19832 from fixed-bed reactor for denitrification of drinking water	397	0.932
		8	32	O: *Rhodobacterales*	F: *Hyphomonadaceae*	AF236001	*Alphaproteobacterium* clone A0904	304	0.776
		3	12	O: *Burkholderiales*	F: *Comamonadaceae*	DQ413087	Uncultured bacterium clone 28 from anaerobic-aerobic SBR	375	0.940
193		22	58	O: *Burkholderiales*	G: *Acidovorax*	EU037281	*Acidovorax* sp. G3DM-41 from soil polluted with chromium	361	0.950
		4	10	O: *Burkholderiales*	G: *Simplicispira*	AJ505861	*Comamonadaceae* bacterium PIV-16-2 from garden soil	352	0.936
198		5	83	O: *Myxococcales*		DQ088736	Uncultured bacterium clone BE16FW031601GDW_hole1-3 from gold mine	265	0.923
202		2	100	O: *Rhodocyclales*	G: *Rhodocyclus*	AF204247	Uncultured *Betaproteobacterium* SBRB34 from EBPR sludge	315	0.812
212		47	33	O: *Burkholderiales*		DQ066963	Uncultured bacterium clone pLW-2 from Lake Washington sediments	376	0.913
		31	22	O: *Burkholderiales*	G: *Mitsuaria*	AB240354	Uncultured bacterium from rhizosphere	376	0.826
		13	11	O: *Rhodocyclales*	G: *Rhodocyclus*	EF565152<br>AB276369	Uncultured bacterium clone VIR_A1 from EBPR sludge enriched in *Accumulibacter*<br>Uncultured *Betaproteobacterial* clone sequence with regions of perfect match with PAOmix	356	0.944
		10	7	O: *Burkholderiales*	G: *Xenophilus*	EF125952	Uncultured *Variovorax* sp. clone B5 from biofilm-reactor based enrichment of bacteria utilizing acyl-homoserine lactones	307	0.812
213		283	98	O: *Burkholderiales*	G: *Paucibacter*	FJ535228	Uncultured *Gammaproteobacterium* clone ATB-LH-7199 from carrot wash water	330	0.699
214		1254	74	O: *Rhodocyclales*	G: *Rhodocyclus*	AB200295<br>AF502230	Uncultured bacterium clone UTFS-OF09-d22-93 from EBPR sludge<br>Uncultured bacterium clone HP1A31 from EBPR sludge	422	0.898
		195	12	O: *Rhodocyclales*	G: *Dechloromonas*	AY032611	*Dechloromonas* sp. JJ oxidizing benzene coupled with nitrate reduction in pure culture by two strains of *Dechloromonas*	320	0.703
		150	9	C: *Betaproteobacteria*		CU924494	Uncultured *Betaproteobacterium* clone QEDP2DB07 from anaerobic digester	409	0.930
		47	3	O: *Burkholderiales*	G: *Rhodoferax*	FM955857	*Rhodoferax* sp. Asd M2A1 from melt water stream from an Arctic glacier	340	0.885
215		26	84	O: *Rhodocyclales*	G: *Rhodocyclus*	EF590005	Uncultured bacterium clone D24 from nitrobenzene polluted river	366	0.853
		2	6.5	O: *Burkholderiales*	F: *Comamonadaceae*	EU180529	*Betaproteobacterium* clone BAC49 from granular activated carbon filters	375	0.895
		2	6.5	C: *Anaerolineae*		EU862289	Uncultured bacterium clone K75 from EBPR sludge	322	0.882
		1	3	O: *Rhodocyclales*	G: *Dechloromonas*	AY064177	Uncultured *Betaproteobacterium* clone UCT N123 from EBPR-WWTP	304	0.916
216		6	46	O: *Rhodocyclales*	G: *Rhodocyclus*	CU920179	Uncultured *Betaproteobacterium* clone QEDT2AF08 from anaerobic digester	352	0.946
		4	31	O: *Burkholderiales*	F: *Comamonadaceae*	AB475015	Uncultured *Betaproteobacterium* clone B20_FuJY from iron containing freshwater	331	0.895
217		5	100	O: *Rhodocyclales*	G: *Rhodocyclus*	AY913841	Uncultured bacterium clone MBR283-54 from H_2-fed fixed-film bioreactor	344	0.843
220		10	67	O: *Phycisphaerales*		AY957928	Uncultured bacterium clone B3NR69D13 from drinking water biofilms	191	0.468
		4	27	O: *Rhizobiales*	G: *Aminobacter*	NR025302	*Aminobacter niigataensis* strain DSM7050	333	0.925
222		213	98	O: *Rhodobacterales*	F: *Hyphomonadaceae*	AF236001<br>AJ227809	Uncultured *Alphaproteobacterium* clone A0904<br>Maricaulis sp.	284	0.638
		4	2	O: *Sphingomonadales*	G: *Novosphingobium*	CU919596	Uncultured *Alphaproteobacterium* clone QEDV3CA09 from anaerobic digester	367	0.979

Table SM5.2 – *Continued*

T-RF[1] (bp)	Sample[2]	Counts[3] (-)	Fraction of T-RF[4] (%)	Affiliation[5] Phylum → Order	Family → Genus	Accession number[5]	Closest relative and original microbiota[6]	Smith-Waterman mapping score (-)[7] Absolute	Normalized
224	PAO109	16	84	O: *Actinomycetales*	F: *Intrasporangiaceae*	AF255629	Uncultured bacterium clone Ebpr19 from EBPR sludge	365	0.818
		3	16	O: *Desulfuromonadales*		DQ309326	*Desulfuromonas alkaliphilus* strain Z-0531	172	0.379
227		13	100	O: *Actinomycetales*	F: *Microbacteriaceae*	CU922723	Uncultured *Actinobacterium* clone QEDR3AC10 from anaerobic digester	381	0.923
238		13	93	P: *Armatimonadetes*		DQ975217	Uncultured bacterium clone vf23 from freshwater wetland	213	0.552
251		3	100	O: *Spirochaetales*	G: *Spirochaeta*	AJ565434	*Spirochaeta* sp. MWH-HuW24 isolated from freshwater pond	300	0.896
254		17	95	O: *Sphingobacteriales*		DQ413096	Uncultured bacterium clone 37 from anaerobic-aerobic SBR	346	0.885
255		16	100	O: *Sphingobacteriales*		FM872812	Uncultured bacterium clone FB03C04 from house dust	331	0.834
257		12	100	O: *Sphingobacteriales*		EU104313	Uncultured bacterium clone N1903_57 from activated sludge	345	0.876
258		6	100	O: *Sphingobacteriales*		EU104041	Uncultured bacterium clone M0111_42 from activated sludge	341	0.909
260		6	100	O: *Sphingobacteriales*		EU104185	Uncultured bacterium clone N1512_12 from activated sludge	214	0.728
277		4	100	P: *Spirochaetes*	F: *Leptospiraceae*	AY293856	*Turneriella parva serovar* Parva strain H	381	0.828
286		2	100	O: *Rhizobiales*	G: *Devosia*	CU926373	Uncultured *Alphaproteobacterium* clone QEDN4AH09	319	0.853
288		6	46	O: *Sphingomonadales*	G: *Sphingopyxis*	AJ416410	*Sphingopyxis witflariensis* isolated from activated sludge	395	0.884
		5	39	O: *Sphingomonadales*	G: *Sphingosinicella*	EF363041	Uncultured bacterium clone R1-10 from deep saline fluid from continental drilling hole	351	0.949
289		10	100	O: *Sphingomonadales*	G: *Sphingopyxis*	EF424392	*Sphingopyxis* sp. P27 from soil contaminated by hexachlorocyclohexane	394	0.900
295		11	100	P: *Chloroflexi*	G: *Herpetosiphon*	CP000875	*Herpetosiphon aurantiacus* DSM 785	271	0.763
297		114	100	P: *Chloroflexi*	G: *Herpetosiphon*	NC009972	*Herpetosiphon aurantiacus* DSM 785	394	0.851
298		221	99.5	P: *Chloroflexi*	G: *Herpetosiphon*	CP000875	*Herpetosiphon aurantiacus* DSM 785	369	0.795
303		8	89	O: *Sphingobacteriales*	F: *Flexibacteraceae*	AY854022	*Leadbetterella byssophila* strain 4M15	239	0.658
		1	11	C: *Gammaproteobacteria*		FJ356049	Uncultured bacterium clone G5 from EBPR reactor	304	0.840
306		45	100	P: *Armatimonadetes*		CU921283	Uncultured unclassified bacterium clone QEDS2BB03 from anaerobic digester	196	0.417
314		6	100	P: *Chloroflexi*	G: *Herpetosiphon*	NC009972	*Herpetosiphon aurantiacus* DSM 785	259	0.685
321		13	93	O: *Sphingobacteriales*		AF368190	Uncultured *Haliscomenobacter* sp. clone SBRT303 from non-EBPR sludge	261	0.676
393		26	100	C: *Deltaproteobacteria*	G: *Bdellovibrio*	CU466777	Uncultured bacterium sequence from anoxic basin of a municipal WWTP	273	0.613

[1] Size of target terminal-restriction fragments (T-RF) obtained with *Hae*III digestion and forming operational taxonomic units (OTU).
[2] Original grab biomass sample taken on day 109 in the PAO-SBR for pyrosequencing analysis.
[3] Number of sequences from the pyrosequencing dataset that were related to the particular reference organism.
[4] Different bacterial populations can contribute to the same T-RF. The percentage of contribution of each population to the target T-RF is given in this column.
[5] Closest bacterial affiliations and GenBank accession numbers obtained after mapping against the Greengenes reference sequences of 16S rRNA encoding gene (McDonald *et al.*, 2012). Predominant OTUs present in the bacterial community are highlighted in bold font. Legend: P = phylum, C = class, O = order, F = family, G: genus.
[6] Description of closest relatives and original microbiota from which the reference clones were isolated were obtained after submitting the accession numbers into the GenBank public database (Benson *et al.*, 2011).
[7] The Smith-Waterman (SW) score was used as mapping similarity measure. SW scores consider nucleotide positions and gaps in the sequence structures. The highest absolute SW score that can be obtained is the length of the sequence itself. Each absolute SW score was normalized to the length of the related denoised centroid sequence in order to allow comparison between sequences of various lengths. After mapping in MG-RAST (Meyer *et al.*, 2008), the affiliations obtained for the two denoised pyrosequencing datasets were related to traditional sequence identity scores of 99.7±0.5%.

Table SM5.3 Closest phylogenetic bacterial affiliations of OTUs detected in the **GAO-SBR** obtained with PyroTRF-ID.

T-RF[1] (bp)	Sample[2]	Counts[3] (-)	Fraction of T-RF[4] (%)	Affiliation[5] Phylum → Order	Family → Genus	Accession number[5]	Closest relative and original microbiota[6]	Smith-Waterman mapping score (-)[7] Absolute	Normalized
32	GAO398	40	24	O: *Rhodobacterales*	G: *Thioclava*	AB079681	*Rhodobacter* sp. AP-10	373	0.829
		30	18	C: *Gammaproteobacteria*	F: *Xanthomonadaceae*	EU834776	Uncultured bacterium clone L68 from activated sludge	342	0.924
		28	17	P: *Bacteroidetes*	F: *Flavobacteriaceae*	GQ089203	Uncultured bacterium clone nbw318h06c1 from human skin microbiome	299	0.799
		26	16	O: *Rhizobiales*	G: *Methylocella*	CP001280	*Methylocella silvestris* BL2, an aerobic facultative methanotroph	344	0.804
		9	5	O: *Rhizobiales*	F: *Hyphomicrobiaceae*	AM411913	*Hyphomicrobium* sp. P-47 isolated from soil	367	0.979
		5	3	O: *Rhodobacterales*	G: *Rhodobacter*	FN428770	Uncultured bacterium from Mahananda river, India	289	0.658
		5	3	O: *Sphingobacteriales*	G: *Spirosoma*	EU370956	*Spirosoma panaciterrae* strain Gsoil 1519 isolated from soil	351	0.931
63		8	100	O: *Actinomycetales*	F: *Nocardiaceae*	AF210769	*Nocardioides* sp. CF8, an alkane-utilizing bacterium	299	0.644
178		1099	99.5	C: *Alphaproteobacteria*	F: *Rhodospirillaceae*	AY351639	Uncultured bacterium clone TFOa43 forming tetrads in anaerobic-aerobic processes	397	0.865
		6	0.5	C: *Acidobacteria*	F: *Acidobacteriaceae*	AY326572	Uncultured soil bacterium clone 1031-2 from terra preta and pristine forest Amazon soil	257	0.562
184		11	100	O: *Rhizobiales*	G: *Methylocystis*	AJ868421	*Methylocystis* sp. 5FB1 from a methanotrophic consortium	289	0.691
186		5	45	O: *Rhizobiales*	F: *Bradyrhizobiaceae*	AY345540	Unidentified bacterium clone LWSR-28 from the Hawaiian Archipelago	327	0.811
		4	36	C: *Alphaproteobacteria*	F: *Rhodospirillaceae*	AY351640	Uncultured bacterium clone TFOa44 forming tetrads in anaerobic-aerobic processes	317	0.722
		2	18	O: *Rhizobiales*	G: *Rhodoplanes*	CU918797	Uncultured *Alphaproteobacterium* clone QEEA3DF04 from anaerobic digester	346	0.989
187		48	87	O: *Rhodobacterales*	F: *Hyphomonadaceae*	AF236001	*Alphaproteobacterium* A0904 sequence	293	0.757
		7	13	O: *Rhizobiales*	F: *Bradyrhizobiaceae*	AF502220	Uncultured bacterium clone HP1B78 from EBPR sludge	368	0.882
190		17	52	O: *Rhizobiales*		GU455290	Uncultured bacterium clone thermophilic_alkaline-50 from process of alkaline fermentation of waste activated sludge producing short-chain fatty acids	354	0.881
		14	42	O: *Rhizobiales*	F: *Bradyrhizobiaceae*	AF208509	*Bradyrhizobium japonicum* strain USDA 129	351	0.900
194		215	100	C: *Alphaproteobacteria*	F: *Rhodospirillaceae*	AF527585	Uncultured bacterium clone LPB46 from EBPR sludge	420	0.915
200		18	86	C: *Acidobacteria*	F: *Acidobacteriaceae*	FJ466403	Uncultured bacterium clone E201 from CO-oxidizing consortium on Hawaiian volcanic deposit	320	0.775
		3	14	P: candidate phylum TM7		EU135398	Uncultured bacterium clone FFCH4275 from soil	251	0.693
208		22	55	O: *Burkholderiales*		EU491323	Uncultured bacterium clone P9X2b8C04 from ocean crust of Loi'hi seamount	364	0.901
		17	43	C: *Acidobacteria*	F: *Acidobacteriaceae*	EU445233	Uncultured bacterium clone R93 from tropical forest topsoil	293	0.753
209		4009	90	C: *Acidobacteria*	F: *Acidobacteriaceae*	AF200696	Uncultured *Acidobacterium* UA1 from 13C-methanol exposed soil	418	0.897
		427	10	P: *Armatimonadetes*		GQ264378	Uncultured bacterium clone WW2_58 from simulated low-level-radioactive-waste site	193	0.410
210		23	72	C: *Acidobacteria*	F: *Acidobacteriaceae*	AF200696	Uncultured *Acidobacterium* UA1 from 13C-methanol exposed soil	277	0.655
211		15	79	P: *Armatimonadetes*		GQ264378	Uncultured bacterium clone WW2_58 from simulated low-level-radioactive-waste site	189	0.512
		3	9	C: *Acidobacteria*	F: *Candidatus* Solibacter	DQ404599	Uncultured bacterium clone 656043 from contaminated sediments	342	0.851
		3	9	O: *Rhodobacterales*	F: *Hyphomonadaceae*	EU770258	*Alphaproteobacterium* A4 sequence from a colony-associated bacteria of the cyanobacterium *Microcystis aeruginosa*	166	0.401
212		21	78	O: *Rhodocyclales*	F: *Rhodocyclaceae*	DQ664245	*Betaproteobacterium* clone IMCC1729 from freshwater pond	431	0.947
		3	11	O: *Hydrogenophilales*	G: *Thiobacillus*	AY955087	Uncultured bacterium clone 5-10m from sediments of the Guanting Reservoir	199	0.435
216		15	100	O: *Hydrogenophilales*	G: *Thiobacillus*	FM212998	Uncultured bacterium clone H2SRC219 from biotrickling filter removing H_2S	400	0.871
220		3	75	O: *Rhizobiales*	G: *Mezorhizobium*	GQ221761	*Prosthecomicrobium enhydrum* strain 9b	356	0.978
222		18	100	O: *Sphingomonadales*		AJ746092	*Novosphingobium* sp. MG35 isolated from haemodialysis water distribution	379	0.920
223		7	35	O: *Actinomycetales*	G: *Tetrasphaera*	DQ007320	*Tetrasphaera vanveenii* strain Ben 70	309	0.655
		11	55	O: *Actinomycetales*	G: *Propionicimonas*	FM178834	Uncultured bacterium clone 672_O24_PCE_column_outflow from aquifer soil column degrading PCE	393	0.854
235		8	100	C: *Gammaproteobacteria*		FJ356048	Uncultured bacterium clone C90 from anaerobic-aerobic lab-scale EBPR system	276	0.860
237		63	97	C: *Gammaproteobacteria*		EU529737	Uncultured bacterium clone H96 from EBPR system	395	0.862
238		3910	100	C: *Gammaproteobacteria*		AY098896	Uncultured proteobacterium clone SBRL1_8 from anaerobic-oxic activated sludge process	431	0.917
239-248		116	100	C: *Gammaproteobacteria*		AY098896	Uncultured proteobacterium clone SBRL1_8 from anaerobic-oxic activated sludge process	326	0.721

Table SM5.3 – *Continued*

T-RF[1] (bp)	Sample[2]	Counts[3] (-)	Fraction of T-RF[4] (%)	Affiliation[5] Phylum → Order	Family → Genus	Accession number[5]	Closest relative and original microbiota[6]	Smith-Waterman mapping score (-)[7] Absolute	Normalized
250	GAO398	2	100	C: *Gammaproteobacteria*	G: *Rhodanobacter*	FJ536898	Uncultured *Xanthomonadaceae* bacterium clone DMS28 from municipal WWTP	356	0.906
253		219	100	O: *Sphingobacteriales*		DQ984594	Uncultured bacterium clone IYF104 from Mt. Nan-Jen litterfall	304	0.654
255		19	100	O: *Sphingobacteriales*		EF018676	Uncultured Bacteroidetes bacterium clone Amb_16S_965 from Aspen rhizosphere	287	0.664
256		326	100	O: *Sphingobacteriales*		GQ396989	Uncultured bacterium clone AK1DE1_08G from deglaciated soil	294	0.681
286		71	99	O: *Rhizobiales*	G: *Bradyrhizobium*	FJ192733	Uncultured *Bradyrhizobium* sp. clone GI5-005-F05 from spacecraft assembly clean	275	0.611
288		175	100	O: *Sphingomonadales*	G: *Sphingomonas*	EU133552	Uncultured bacterium clone FFCH3058	395	0.868
290		17	100	C: *Alphaproteobacteria*	F: *Rhodospirillaceae*	AM411928	*Alphaproteobacterium* P-61 from rice paddy soil microcosm	365	0.919
303		123	100	C: *Gammaproteobacteria*		AY098896	Uncultured proteobacterium clone SBRL1_8 from anaerobic-oxic activated sludge process	310	0.797
321		10	100	O: *Sphingobacteriales*		DQ984594	Uncultured bacterium clone IYF104 from Mt. Nan-Jen litterfall	281	0.739
404		8	100	O: *Planctomycetales*	G: *Planctomyces*	CU926004	Uncultured *Planctomycetes* bacterium clone QEDN5CA12 from anaerobic digester	209	0.522
408		7	100	C: *Gammaproteobacteria*	G:*Pseudoxanthomonas*	DQ984530	Uncultured bacterium clone FD_1 16S from oil-contaminated soil	372	0.795

[1] Size of target terminal-restriction fragments (T-RF) obtained with *Hae*III digestion and forming operational taxonomic units (OTU).
[2] Original grab biomass sample taken on day 398 in the GAO-SBR for pyrosequencing analysis.
[3] Number of sequences from the pyrosequencing dataset that were related to the particular reference organism.
[4] Different bacterial populations can contribute to the same T-RF. The percentage of contribution of each population to the target T-RF is given in this column.
[5] Closest bacterial affiliations and GenBank accession numbers obtained after mapping against the Greengenes reference sequences of 16S rRNA encoding gene (McDonald *et al.*, 2012). Predominant OTUs present in the bacterial community are highlighted in bold font. Legend: P = phylum, C = class, O = order, F = family, G: genus.
[6] Description of closest relatives and original microbiota from which the reference clones were isolated were obtained after submitting the accession numbers into the GenBank public database (Benson *et al.*, 2011).
[7] The Smith-Waterman (SW) score was used as mapping similarity measure. SW scores consider nucleotide positions and gaps in the sequence structures. The highest absolute SW score that can be obtained is the length of the sequence itself. Each absolute SW score was normalized to the length of the related denoised centroid sequence in order to allow comparison between sequences of various lengths. After mapping in MG-RAST (Meyer *et al.*, 2008), the affiliations obtained for the two denoised pyrosequencing datasets were related to traditional sequence identity scores of 99.7±0.5%.

Supplementary material 6

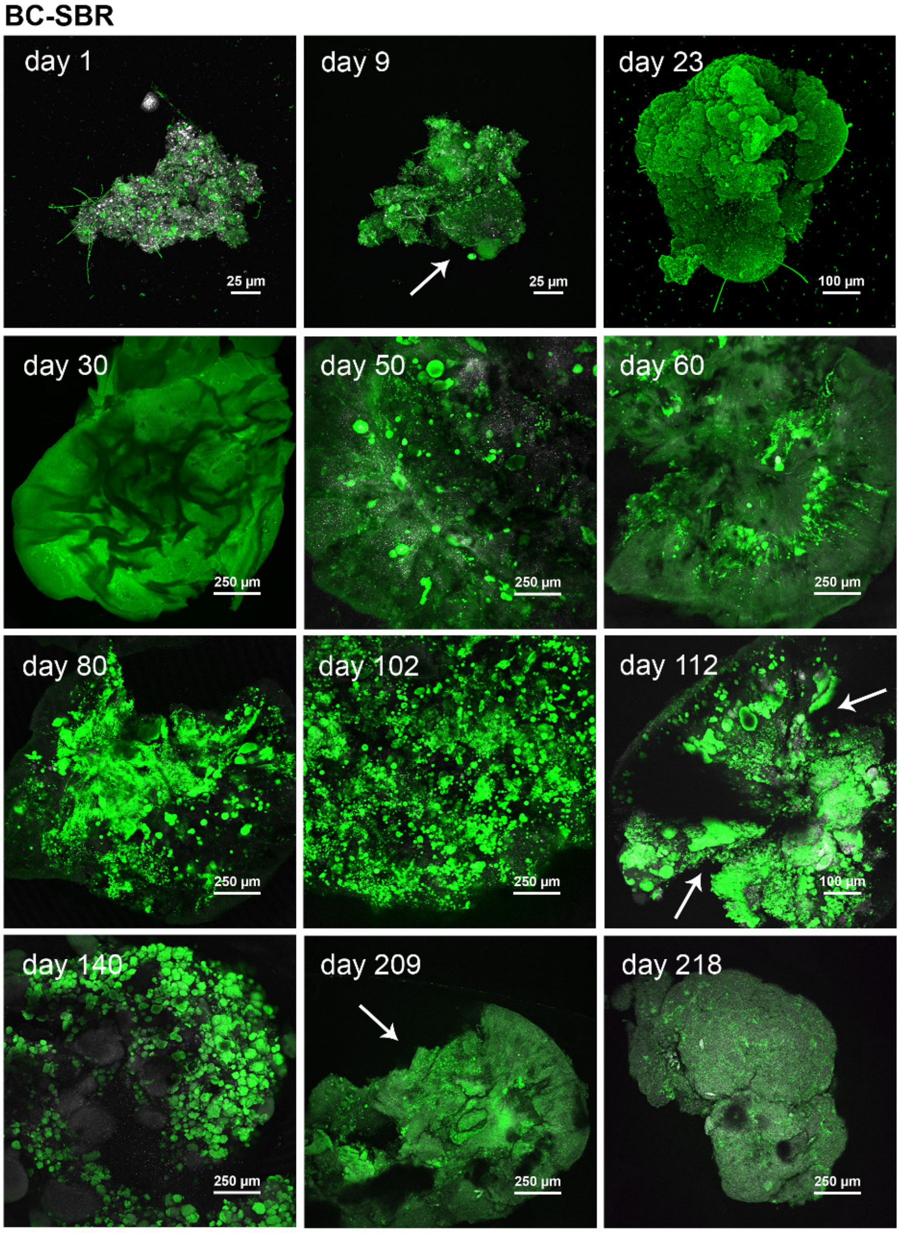

Figure SM6.1 Structural dynamics during granule formation and maturation in the BC-SBR.

Temporal evolution of the architecture of bioaggregates from activated sludge flocs (day 1) to early-stage (day 23) and mature granular biofilms (day 102) in the BC-SBR operated under wash-out conditions. CLSM datasets were recorded on full bioaggregates from samples taken from day 1 to day 23, as well as on day 218. Granules from samples taken between day 30-140 were analyzed on cross-sections. The sample taken on day 209 was analyzed as 80-µm cryosection. The green fluorescent dye Rhodamine 6G was used to map cells and biofilm matrices. In 8 bit data sets, 256 green levels were allocated to this dye. The reflection signal was used as reference with 256 grey/white color allocation. On day 9, swelling of microbial colonies around the floc structure can be observed. Early-stage granule nuclei on day 23 were 4-5 times bigger than flocs, and displayed compact biofilm aggregation. On day 30, early-stage granules were composed of a continuous biofilm displaying homogenous cell distribution and folded structures. From day 30 to day 102, the internal architecture of granules evolved with the proliferation of dense microcolonies from the granule core outwards. Larger microbial clusters appeared in the structure of granules between day 112 and 140. Detachment phenomena contributed to the heterogeneous structure of mature granules (days 112 and 209). After more than 200 days, mature granules exhibited aggregation of dense biofilm clusters, internal voids, and eroded surface slimy structures.

Supplementary material 7

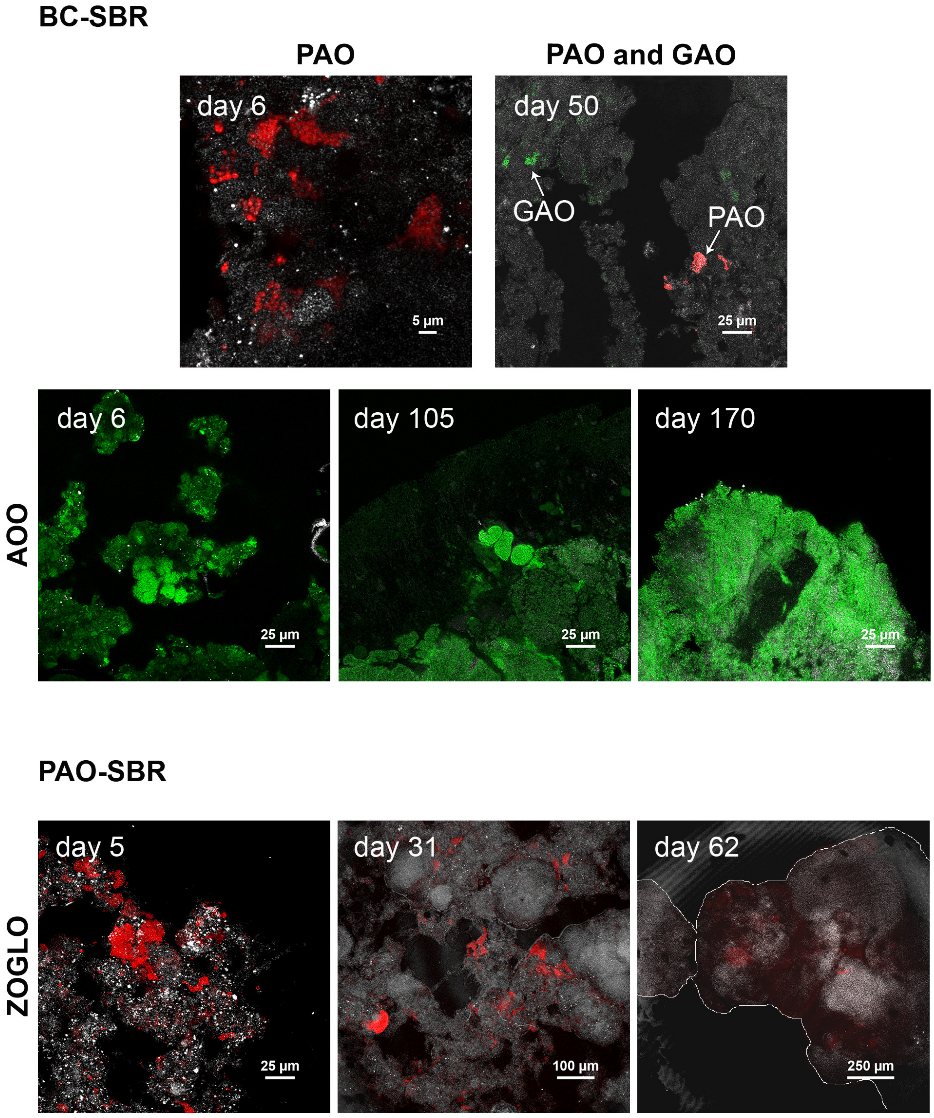

Figure SM7.1 Spatial dynamics of additional bacterial populations during granule formation and maturation in the BC-SBR and PAO-SBR.

The FISH-CLSM analyses revealed that *Accumulibacter* affiliates (PAO, red) were initially present in the flocculent activated sludge on day 6 in the BC-SBR. These organisms and their competitors affiliating with *Competibacter* (GAO, green) were only present in low abundances on day 50, and were outcompeted by *Zoogloea* spp. (Fig. 6). Ammonium-oxidizing organisms (AOO, green) were detected on day 6 inside flocs. During AGS maturation, AOO proliferated across granules as dense microcolony clusters (day 105), and form wider population matrices near edges of mature granules (day 170). In the PAO-SBR, *Zoogloea* spp. (ZOGLO, red) were never abundant during granulation in the PAO-SBR.

Supplementary material 8

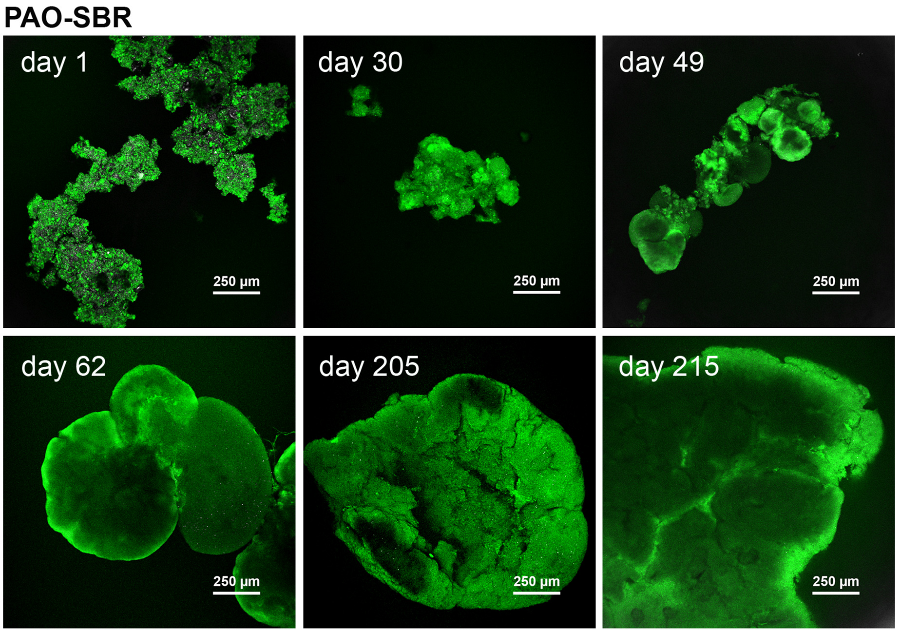

Figure SM8.1 Temporal evolution of the architecture of bioaggregates from flocs to early-stage granules up to mature granules in the stirred-tank PAO-SBR operated under steady-state conditions.

Granule formation occurred in the PAO-SBR by smothering of flocs (day 30) and proliferation of dense microbial clusters around the floc structure (day 49). Dense granule nuclei obtained after 62 days evolved towards mature granules exhibiting folded biofilm structures (day 205) and aggregation of big, dense and homogeneous clusters (day 215).

Supplementary material 9

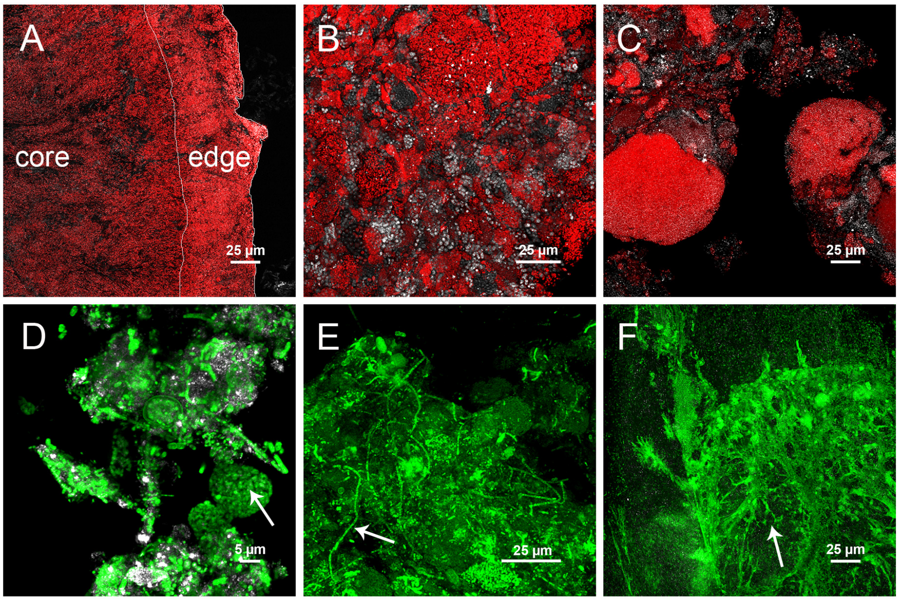

Figure SM9.1 Additional structural features of bioaggregates detected during granulation.

The cross-section of an early-stage granule collected on day 50 in the BC-SBR and hybridized with *Zoogloea*-targeted gene probes **(A)** revealed a more homogeneous cell composition than the highly-heterogeneous "bacterial metropolis" of a mature granule cross-section collected on day 111 in the same reactor and stained with SYPRO Red **(B)**. Fig. **A** also provides information on the palisade-like biofilm outgrowth lines, and on the denser cell aggregation on the first 75 µm from the granule edge. The heterogeneous structure of mature granules obtained in the BC-SBR, that is actually dominated by PAO on day 111, is similar to the one of granules cultivated in the PAO-SBR after 31 days **(C**, color allocation: red = PAO gene probe**)**. Further structures were detected by staining with Rhodamine 6G, such as the globular microcolonies swelling around flocs after 6 days in the BC-SBR **(D)**, filamentous populations detected in the bioaggregates of the PAO-SBR after day 11 **(E)**, and slimy structures at the surface of mature granules collected after 209 days in the BC-SBR **(F)**.

Supplementary material 10

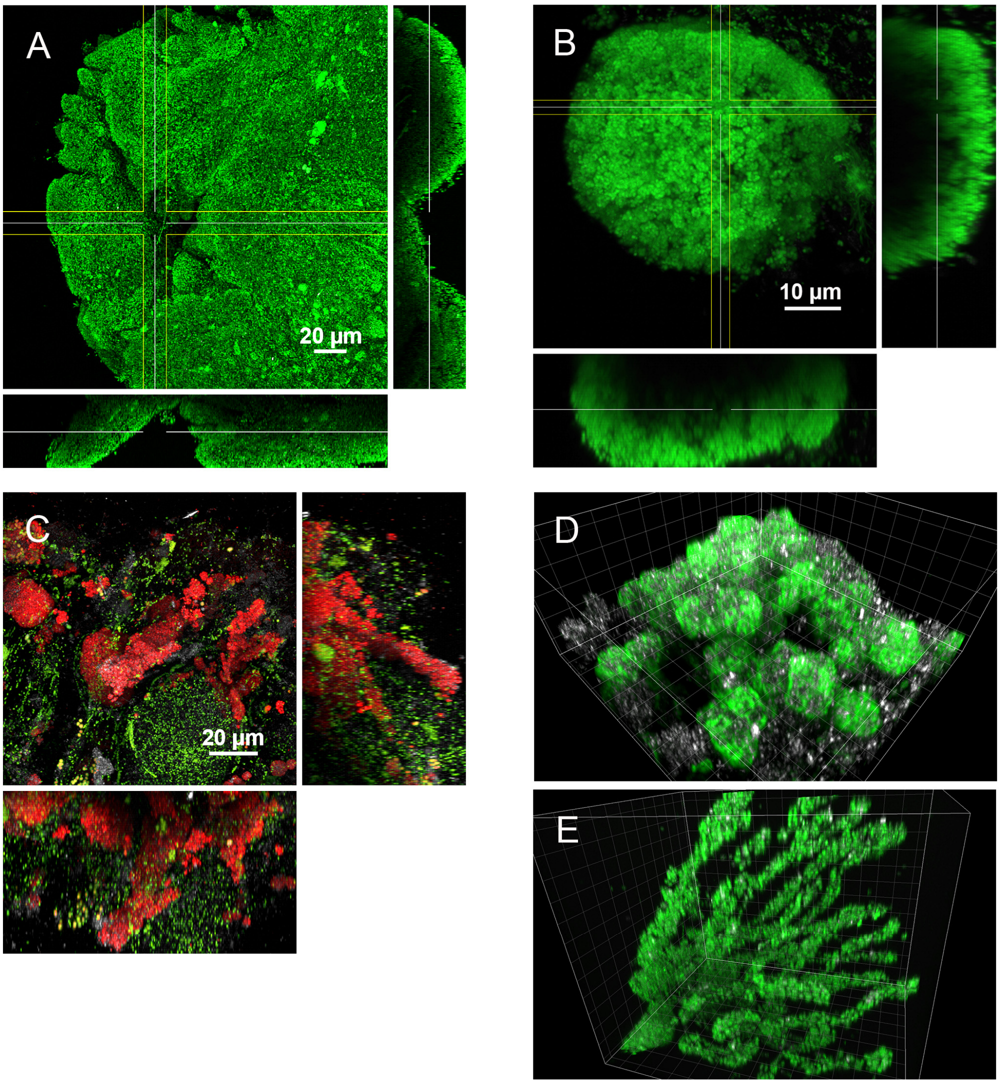

Figure SM 10.1 Three-dimensional examinations of specific structures of granular biofilms.

The 3-D analyzes were conducted with XYZ projections **(A-C)** and 3-D volume projection **(D-E)**. The smooth early-stage granular biofilms on day 20 in the BC-SBR exhibited a surface roughness with biofilm protuberances and valleys **(A)**. A zoom on a spherical microcolony in a mature granule on day 111 in the BC-SBR revealed denser cell aggregation at the edge of the colony **(B)**. Dual staining with SYTOX Green (nucleic acids) and Nile Red (hydrophobic cellular components and poly-β-hydroxyalcanoates) revealed different cell types in the heterogeneous structure of mature granular biofilms on day 111 in the BC-SBR **(C)**. The WGA lectin highlights the 3-D distribution of glycoconjugate matrices surrounding interfaces of microcolony clusters **(D**, grid size of 5 µm**)**. Finger-like zoogloeal population structures were detected in the architecture of aggregates collected on day 15 in the BC-SBR **(E**, grid size of 5 µm**)**. Color allocation in fig. **A**, **B** and **E**: Rhodamine 6G (green) and reflection (gray/white).
